# Application of Pulsed Laser Deposition (PLD) Technology in the Preparation of Two-Dimensional (2D) Film Materials

**DOI:** 10.3390/ma18132999

**Published:** 2025-06-24

**Authors:** Jixiang Cai, Feixing Li, Xueshuai Zhang, Jianguo Wang, Zecong Yu, Bo Feng, Youwen Li

**Affiliations:** 1 Xinjiang Biomass Solid Waste Resources Technology and Engineering Center, College of Chemistry and Environmental Science, Kashi University, Kashi 844000, China; caijixiangedukashi@163.com (J.C.);; 2 Shaanxi Key Laboratory of Condensed Matter Structures and Properties and MOE Key Laboratory of Materials Physics and Chemistry Under Extraordinary Conditions, School of Physical Science and Technology, Northwestern Polytechnical University, Xi’an 710072, China

**Keywords:** pulsed laser deposition (PLD), two-dimensional (2D) film materials, application

## Abstract

Two-dimensional film materials with unique atomic structures and electronic operation modes have demonstrated amazing application potential and value in the field of high technology. Among the various methods for preparing 2D film materials, PLD technology has become the preferred technology for rapid and green preparation of high-quality, complex structured 2D film materials due to its features such as maintaining the excellent stoichiometric ratio of the target, strong process flexibility, and non-polluting environment. Therefore, this paper discusses the exciting topic of PLD technology in the preparation and application of 2D film materials. Based on a systematic exposition of its basic principles and influencing factors, it provides a detailed overview of the current application status of PLD technology in the preparation of various 2D film materials such as carbides, sulfides, oxides, nitrides, and perovskites. Meanwhile, the advantages and disadvantages of PLD technology in the preparation of 2D film materials were also positively summarized, and the challenges and emerging strategies it faces in the future preparation of 2D film materials were cautiously discussed. This provides practical suggestions and reflections for the sustainable development of PLD technology in the fields of basic research, performance regulation, device development, and application of 2D film materials preparation.

## 1. Introduction

Materials play an important role that cannot be ignored in the development of human society and the progress of science and technology [1]. As the cornerstone of human civilization, the use of materials is an important symbol that distinguishes humans from other animals, and the emergence of each new material is driving humanity into a new stage of civilization development [2]. Meanwhile, materials are also the foundation of modern industry, and their level of development directly affects the competitiveness and economic benefits of various industries, thus having a profound impact on the development of the global economy and society [3]. In addition, the continuous innovation of materials is actively promoting social reform and technological progress while providing people with more diverse and convenient products [4]. Therefore, the development and innovation of materials are crucial in meeting people’s expectations for a cleaner, more convenient, and better life.

Generally speaking, materials can be classified into zero-dimensional (0D) materials, one-dimensional (1D) materials, 2D film materials, and three-dimensional (3D) materials according to different dimensions [5]. Among them, 0D materials refer to materials that are all at the nanoscale (1–100 nm) in three-dimensional space, also known as quantum dots, which have significant quantum size effects, quantum tunneling effects, and unique optical properties [6], but they also have problems such as poor stability, preparation and separation difficulty, easy agglomeration, and so on, which limit their application scope [7]. One-dimensional materials refer to materials with two dimensions at the nanoscale (1–100 nm), while the other dimension is at the macroscopic scale (such as carbon nanotubes, nanowires, and nanorods), which electrons can only move freely in one direction and have excellent electrical properties, adjustable optical properties, and high mechanical strength [8]. At the same time, the complexity of the preparation process, the limited scale of application, and the poor stability of material properties cannot be ignored. Two-dimensional film materials are materials in which electrons can move freely in only two dimensions at non-nanometer scales, and their thickness is usually only a single or a few atomic layers (such as graphene [9], molybdenum disulfide [10], boron nitride [11], etc.). At the same time, they also have unique characteristics such as high carrier mobility, tunable energy band structure, saturable absorption properties, excellent mechanical properties, and high specific surface area and surface activity [12]. Three-dimensional materials refer to materials with macroscopic dimensions (usually greater than 100 nm) in all three dimensions of space, which have the advantages of high structural and performance stability, diverse properties, and mature design and preparation technologies [13], but the difficulty in regulating microscopic properties, limited integration and miniaturization, and the interface and surface effects are not prominent, and other issues have become an important factor restricting the development of their innovations [14]. Among these materials with different dimensions, 2D film materials have unique electrical, optical, magnetic, and other physical properties that are different from other dimensional materials due to their special atomic layer structure and electron motion mode, such as the room temperature quantum Hall effect [15], anomalous superconductivity phenomenon [16], and piezoelectric properties [17], which have a wide range of applications in the fields of optoelectronics [18], biomedicine [19], catalysis [20], and sensors [21] and have received widespread attention.

Since Novoselov et al. [22] successfully separated single-layer graphene from graphite using mechanical exfoliation in 2004, 2D film materials have sparked a worldwide research boom among related researchers in the fields of materials science, physics, and chemistry, which has led to more and more attempts to develop more methods and approaches to prepare 2D film materials with different physicochemical properties [23]. At present, the methods for preparing 2D film materials are mainly categorized into two categories: “top-down” and “bottom-up” [24]. Among them, the top-down method is mainly to peel or decompose the macroscopic bulk material into single-layer or few-layer 2D film materials by physical or chemical means, including the mechanical peeling method [25], the chemical peeling method [26], and the liquid-phase peeling method [27]. The mechanical stripping method is extremely simple to operate, but the yield is extremely low, and the material size and shape are difficult to accurately control [28]. The chemical exfoliation method can achieve large-scale preparation of 2D film materials, but the chemical process may alter the intrinsic properties of the material, thereby affecting its original physical and chemical properties [29]. The liquid-phase exfoliation method is simple to operate, does not involve chemical reactions, and has the potential for large-scale production, but it is difficult to accurately control the number of atomic layers of the material, and the separation and purification process of the products after exfoliation is relatively complex [30]. The bottom-up method is to construct the desired 2D film materials through the gradual assembly of microscopic-scale units such as atoms and molecules, including chemical vapor deposition (CVD) [31], molecular beam epitaxy (MBE) [32], atomic layer deposition (ALD) [33], pulsed laser deposition (PLD) [34], and chemical hydrothermal methods [35]. Compared to the top-down method, the bottom-up method can precisely control the number of layers, the atomic arrangement, and the position and concentration of the dopant atoms of the 2D material from the atomic level, thus achieving precise control of the physicochemical properties of the material [36]. Meanwhile, by selecting different atoms and molecular precursors and designing specific reaction pathways and conditions, 2D film materials with specific functions and structures can be prepared [37]. In addition, the 2D film materials prepared by it have extremely high purity and crystal quality and excellent uniformity and consistency, which makes it more conducive to exerting its intrinsic properties [38]. Among them, PLD technology can precisely control the composition, structure, and growth rate of the film, with strong process flexibility and wide applicability, which plays an important role in the preparation of high-quality, complex-structured 2D film materials [39].

At present, although some review articles on the preparation of 2D film materials have been published, almost all of them focus on traditional methods such as mechanical exfoliation, CVD, and MBE, and there is relatively little review on the preparation of 2D film materials by PLD [40,41,42]. Therefore, this article provides a comprehensive and detailed review on the exciting topic of the preparation of 2D film materials by PLD and their applications. Specifically, this paper first systematically explains the basic principles and influencing factors for the preparation of 2D film materials by PLD. Secondly, a detailed review was conducted on the research status of PLD technology in the preparation of different 2D film materials (including graphene, transition metal sulfides, oxides, perovskites, etc.). Finally, based on the summary of the advantages and disadvantages of PLD technology in the preparation of 2D film materials, the challenges and emerging strategies it faces in the preparation of 2D film materials were seriously discussed, providing a phased summary and reflection for promoting basic research on 2D film materials, facilitating the regulation of 2D material properties and device application development, and expanding the application fields of 2D film materials.

## 2. Basic Principles and Influencing Factors for the Preparation of 2D Film Materials by PLD

### 2.1. Basic Principles for the Preparation of 2D Film Materials by PLD

**Figure 1:**
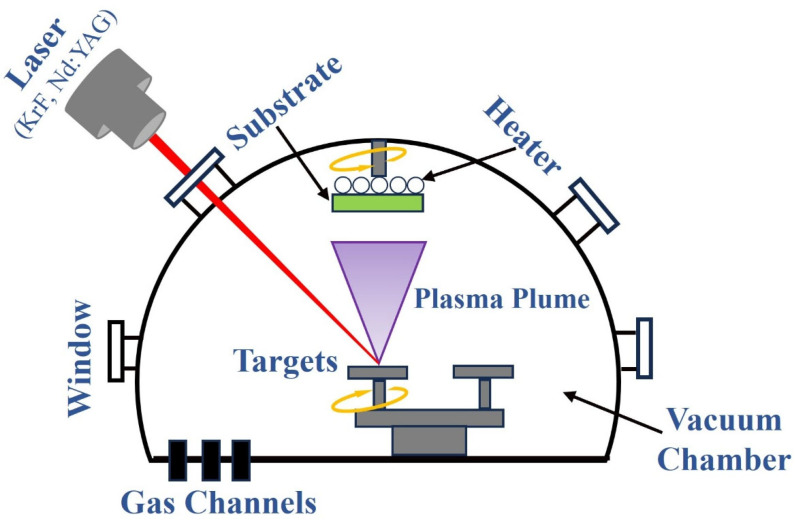
The working principle of preparing 2D film materials by the PLD method.

The working principle of preparing 2D film materials by the PLD method is shown in Figure 1, and its basic process is mainly divided into three stages [43]: (1) The interaction between laser and target material. After the high-energy pulse laser focused by the lens is irradiated onto the surface of the target material, a local area of the target material surface is instantaneously impacted by extremely high energy density, causing the atoms or molecules on the surface of the target material to obtain sufficient energy to be rapidly heated, fused, evaporated, or even ionized, thereby forming a high-temperature, high-density plasma plume. (2) The diffusion and transport of plasma plumes. Plasma plumes containing various particles such as target atoms, ions, electrons, and clusters diffuse and transport towards the substrate with high kinetic energy and velocity in a vacuum or background gas (such as inert gas or reactive gas). (3) Deposition and growth of 2D film materials on the substrate. The particles in the plasma plume diffuse to the surface of the substrate and are adsorbed, deposited, nucleated, and grown, resulting in the formation of 2D film materials with specific compositions and structures.

### 2.2. Influencing Factors for the Preparation of 2D Film Materials by PLD

**Figure 2:**
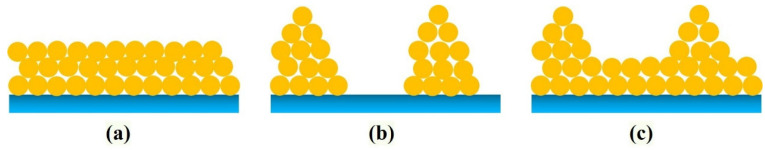
(**a**) Frank–Van der Merwe. (**b**) Volmer Weber or island growth. (**c**) Stranski–Krastanov growth.

It should be noted that many complex physicochemical processes occur during the preparation of 2D film materials by PLD. Among them, the high-energy transient interactions between pulsed lasers of different wavelengths (such as excimer lasers or Nd:YAG lasers) and different targets (metals, inorganic nonmetals, composites, etc.) involve physical theories such as the two-temperature model [44], the thermal conduction model [45], and the phase explosion model [46]. After the plasma plume with high energy density is sputtered out from the surface of the target, intense mutual collisions occur in a limited spatial range and within a very short time, and particles with different energies undergo structural adjustment and redistribution, which is a very complex dynamic process in itself [47]. Finally, when the particles in the plasma plume are sputtered to the surface of the substrate, they adsorb and grow on the substrate [48]. In the adsorption process, the adsorbed atoms diffuse naturally on a homogeneous substrate surface without chemical traps, and their adsorption efficiency depends on the effective coverage of the particles in the plasma plume with the surface of the substrate, but defects in the surface of the substrate can lead to differences in its mass transport [49]. The lattice constant and temperature of the substrate play a decisive role in the growth of the crystal nucleus of 2D film materials during the growth process [50]. Under different growth conditions, it has three different growth patterns [51], as shown in Figure 2. Therefore, during the preparation of 2D film materials by PLD, precise control of factors such as laser parameters, substrate materials, and deposition conditions is of great significance for achieving the regulation of the crystal structure and chemical properties of 2D film materials.

#### 2.2.1. Pulsed Laser Energy

**Figure 3:**
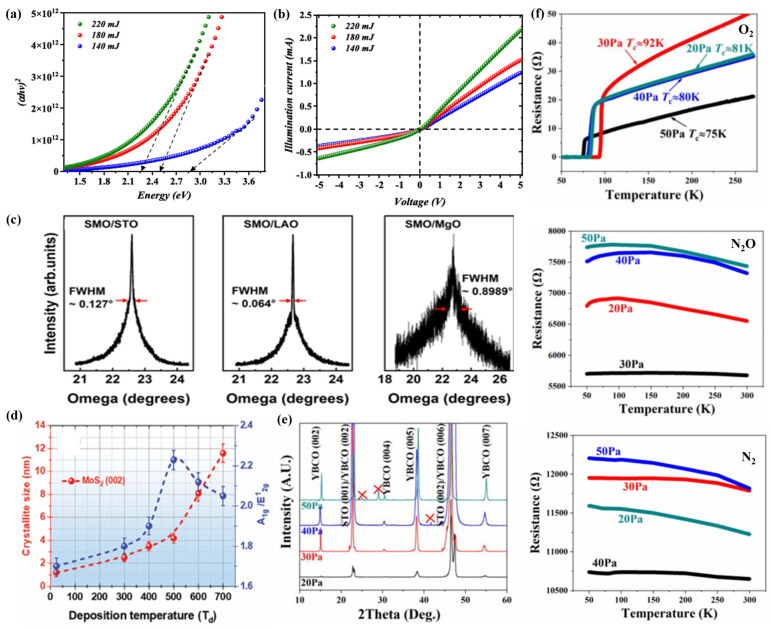
(**a**) Optical bandgap of the deposited SnO_2_:WO_3_ nanofilms at different laser energies. (**b**) The illuminated current–voltage characteristics of the fabricated SnO_2_:WO_3_ photodetectors as a function of laser energy. Reproduced from Ref. [55], Copyright 2024, Published by IOP Publishing Ltd. (**c**) The rocking curves for the (002) diffraction peak of SMO films grown at a base pressure on STO, LAO, and MgO substrates, respectively. Reproduced from Ref. [63], Copyright 2024, American Chemical Society. (**d**) Crystallite size and [A_1g_]/[E^1^_2g_] ratio of MoS_2_ films as a function of deposition temperature. Reproduced from Ref. [67], Copyright 2024, John Wiley and Sons. (**e**) XRD patterns of YBCO films prepared under different pressures in nitrous oxide deposition atmosphere. (**f**) R–T curves of YBCO prepared under different deposition atmospheres and pressures. Reproduced from Ref. [71], Copyright 2023, Elsevier.

In the process of preparing 2D film materials by PLD, the pulsed laser energy is a key factor that directly affects the generation and diffusion of plasma plumes and their deposition, nucleation, and growth on the substrate surface [52]. When the pulsed laser is irradiated on the surface of the target material, if the laser energy is too low, it may not be able to generate plasma plumes, or the generated plasma plumes may not have enough energy to diffuse in the cavity and deposit, nucleate, and grow on the substrate surface [53]. If the laser energy is too high, the plasma plume generated will have high energy, which will cause it to diffuse violently in the cavity and impact strongly on the substrate surface, thus seriously affecting its deposition, nucleation, and growth on the substrate surface [54]. Aldaghri et al. [55] found that, when SnO_2_:WO_3_ nanofilms were prepared by the PLD technique, the grain size, crystallinity, surface roughness, and root mean square of the SnO_2_:WO_3_ film increased with the increase in laser energy, but its bandgap decreased from 2.85 eV to 2.25 eV (Figure 3a). Meanwhile, the performance of the UV photodetectors fabricated with SnO2:WO3 thin films was characterized, and it was found that the electrical performance of the SnO_2_:WO_3_ photodetectors was gradually enhanced with the increase in laser energy (Figure 3b).

#### 2.2.2. Quantity of Laser Irradiation

In the process of preparing 2D film materials using PLD, the thickness of 2D film materials can be effectively controlled by changing the amount of laser irradiation so as to grow high-quality 2D film materials with precisely adjustable thickness [56]. Alvarez et al. [57] used PLD technology to deposit h-BN films with different thicknesses by controlling the number of laser irradiations, and their results showed that, as the thickness of h-BN thin films increased (from 30 nm to 300 nm), the root mean square (RMS) measured with AFM also gradually increased (from 3.30 nm to 17.40 nm), which indicated that the surface roughness of the h-BN films becomes larger, leading to enhanced phonon scattering and a gradual decrease in its cross-sectional thermal conductivity (from 1.5 W/(m k) to 0.2 W/(m k)).

#### 2.2.3. Targets and Substrates

As the basis for ensuring excellent stoichiometry, the physicochemical composition and relative composition of the target are very important for the preparation of high-quality 2D film materials [58]. In the PLD process, elements of different qualities will be deposited on the substrate surface for nucleation and growth at different rates and momenta, resulting in the formation of 2D film materials with different structures and functions [59]. Meanwhile, different kinds of substrates have different crystal structures, crystal plane orientations, and lattice constants, which makes the plasma plume deposition, nucleation, and growth on the substrate become seriously affected [60]. In order to prepare high-quality 2D film materials, lattice mismatch between the target and the substrate is another key factor [61]. Usually, when the lattice mismatch is less than 5%, the 2D film material grows with full coherent epitaxial growth; when the lattice mismatch is between 5% and 25%, the 2D film material grows with incommensurate epitaxial growth; when the lattice mismatch is greater than 25%, the lattice matching ability between the 2D film material and the substrate is lost, thus generating polycrystalline thin film material [62]. Jia et al. [63] used PLD technology to deposit SrMoO_3_ (SMO) films of different thicknesses on (001) SrTiO_3_ (STO), (001) LaAlO_3_ (LAO), and (001) MgO substrates and investigated their effects on the structure and properties, the results of which indicated that SrMoO_4_ films exhibited different crystal orientations on different substrates, and SrMoO_4_ films grew epitaxially along the c-axis on the (001) STO and (001) LAO substrates, while diffraction peaks corresponding to (112) SrMoO_4_ appeared on the (001) MgO substrate, causing SrMoO_4_ films to grow texturally along the c-axis, some parts growing along the (112) axis. At the same time, SMO films grown on different substrates exhibited significant differences in the full width at half-maximum (FWHM) of the (002) diffraction peak, which was mainly caused by the lattice mismatch between the SMO films and the substrates. On the STO and LAO substrates, the lattice mismatch between the SMO film and its substrate is −1.8% and −4.8%, respectively, with FWHM values of 0.127° and 0.064°, respectively, which implies that the SMO film has good crystallinity. On the MgO substrate, the SMO film has a lattice mismatch of +5.7% and a FWHM value of 0.8989°, which is mainly caused by a large number of edge dislocations formed in order to release the strain energy caused by the lattice mismatch (Figure 3c).

#### 2.2.4. Substrate Temperature

The substrate temperature is one of the important factors affecting 2D film materials in PLD technology. At a suitable substrate temperature, the particles deposited onto the substrate can maintain sufficient kinetic energy to rapidly diffuse on the substrate, which is very beneficial for their subsequent nucleation and growth [64]. When the substrate temperature is too low, the particles deposited on the substrate will be quickly deposited near their landing point, resulting in the formation of an amorphous layer [65]. If the substrate temperature is too high, the particles deposited on the substrate will randomly jump and move due to their high energy levels, making them unevenly distributed or forming other cluster structures [66]. Mouloua et al. [67] used the PLD technique to grow MoS_2_ films at different substrate temperatures (25 °C, 500 °C, 600 °C, and 700 °C), and the study showed that the grain size of MoS_2_ 2D films gradually increased with the increase in substrate temperature, while its [A_1g_]/[E^1^_2g_] peak intensity ratio reached a maximum when the substrate temperature reached 500 °C, and the [A_1g_]/[E^1^_2g_] peak intensity ratio started to decrease when the temperature continued to increase (Figure 3d). Meanwhile, the optical bandgap of MoS_2_ films exhibits a substrate temperature dependence opposite to its [A_1g_]/[E^1^_2g_] peak intensity ratio, which decreases gradually with the substrate temperature and reaches a minimum value (1.4 eV) when the substrate temperature is 500 °C, and the optical bandgap starts to increase as the temperature continues to increase, which implies that there is a certain correlation between the optical bandgap of MoS_2_ films and their degree of vertical orientation.

#### 2.2.5. Gas Environment and Pressure

In the process of preparing 2D film materials by PLD, the background gas environment and pressure mainly affect the structure and properties of 2D film materials by participating in both chemical reactivity and regulating the kinetic energy of the plasma plume [68]. On the one hand, 2D film materials such as oxides, carbides, sulfides, and nitrides can be prepared using the PLD process by employing background gases containing elements such as O, C, S, N, and so on [69]. On the other hand, by regulating the background gas pressure, the kinetic energy of the plasma plume can be changed, thereby affecting the crystallinity, surface morphology, and composition of the 2D film materials [70]. Guo et al. [71] used the PLD technique to deposit YBa_2_Cu_3_O_7-δ_ (YBCO) films under different background atmospheres (oxygen, nitrous oxide, and nitrogen) and pressures (20, 30, 40, or 50 Pa), and the study showed that the optimal deposition pressures for growing YBCO films with good c-axis orientation are inconsistent under different background atmospheres during the PLD process. When oxygen and nitrous oxide were used as the background atmosphere, the optimal deposition pressure was 30 Pa, while, under a nitrogen atmosphere, the optimal deposition pressure was 40 Pa (Figure 3e), and the thickness of the YBCO films increased with the increase in deposition pressure. Meanwhile, the YBCO films grown under an oxygen atmosphere all showed excellent superconductivity, which reached the highest superconducting critical transition temperature of 92 K at the deposition pressure of 30 Pa. In contrast, the YBCO films grown under nitrous oxide or nitrogen atmosphere exhibit remarkable semiconducting properties, and their resistance decreases gradually with the increasing temperature (Figure 3f).

## 3. Application of PLD Technology in the Preparation of 2D Film Materials

### 3.1. Application of PLD in Carbides

Carbon, as a widely existing basic element in nature, can be combined with other elements through three hybridized forms, such as sp, sp^2^, and sp^3^, thus giving it different structures and various allotropes [72]. The versatility of these constituent structures makes their carbon-based compounds rich in physical properties such as mechanical, thermodynamic, electrical, and optical, as well as unique chemical properties, making them widely utilized in scientific research and industrial applications [73]. Among them, the use of PLD technology for the preparation and application of 2D carbon-based materials represented by graphene, diamond-like carbon (DLC), and carbon nanotube (sheet) films has attracted widespread attention. Table 1 is the summary of the deposition conditions and properties of carbides by PLD.

#### 3.1.1. Application of PLD in Graphene

As one of the allotropes of carbon, graphene has immeasurable application potential and research value in various fields, such as electronic devices, sensors, and functional composite materials due to its excellent conductivity, thermal conductivity, good transparency, strong mechanical properties, and other physicochemical properties, which have made it one of the hot topics in modern materials science research [89]. Among the various methods for preparing 2D graphene materials, PLD has the advantages of mild reaction conditions, diverse substrate and target types, and controllable film stoichiometry, making it an important way to obtain graphene efficiently and conveniently [90].

**Figure 4:**
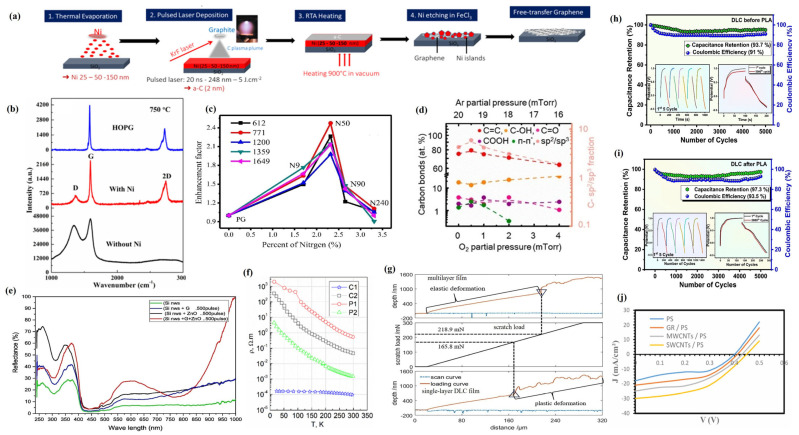
(**a**) Synthesis route for transfer-free graphene films obtained by pulsed laser deposition of carbon on Ni films followed by rapid thermal annealing and Ni etching. Reproduced from Ref. [74], Copyright 2021, Elsevier. (**b**) Raman spectra of graphene grown on glass substrates with and without a Ni catalytic layer. Reproduced from Ref. [75], Copyright 2019, Elsevier. (**c**) Relative enhancement factor of N-doped graphene as a function of the percent of nitrogen. Reproduced from Ref. [78], Copyright 2017, Elsevier. (**d**) Variation of chemical bonds and carbon sp^2^/sp^3^ fraction with respect to growth pressures in the rGO film. Reproduced from Ref. [80], Copyright 2020, American Chemical Society. (**e**) The reflectivity as a function of the wavelength for all the prepared devices. Reproduced from Ref. [81], Copyright 2024, Elsevier. (**f**) Temperature-dependent resistivity characteristics of all the samples. Reproduced from Ref. [83], Copyright 2023, IOP Publishing Ltd. (**g**) Measurement of the critical load. Reproduced from Ref. [84], Copyright 2023, Elsevier. (**h**) Cyclic stability test and coulombic efficiency of pristine DLC film electrode over 5000 cycles. (**i**) Cyclic stability test and coulombic efficiency of a PLA-treated DLC film electrode over 5000 cycles. Reproduced from Ref. [85], Copyright 2024, Elsevier. (**j**) J–V characteristic for porous silicon and a nanocarbon/PS heterojunction solar cell. Reproduced from Ref. [87], Copyright 2020, Trans Tech Publications Ltd.

It is very difficult to directly grow 2D graphene films on insulating substrates using PLD technology, while metal catalysts can effectively promote the growth of graphene films and improve their physical and chemical properties [91]. Nickel–metal catalysts have significant advantages in promoting graphene surface growth and structural optimization on glass substrates due to their high lattice mismatch and high carbon solubility, which have received significant attention [92]. Bleu et al. [74] used PLD technology to prepare 2D graphene films on silica substrates deposited with Ni catalysts (Figure 4a), and their results showed that graphene synthesized on silica substrates with a Ni catalyst thickness of 25 nm had ideal uniformity and low defect density. Among them, a few layers (three to six layers) of graphene dominated, and only 5% of the area was bilayered graphene. After FeCl_3_ treatment, the Ni catalyst was removed, but there was no significant change in the number of graphene layers and structure. Kumar et al. [75] successfully prepared high-quality 2D films of graphene on glass substrates coated with a Ni catalyst, and they found that the samples prepared in the absence of a Ni catalyst had more structural defects and lower crystallinity, and no graphene layers were formed. In contrast, the samples prepared in the presence of a Ni catalyst had high-quality graphene films with high crystallinity (Figure 4b).

The use of PLD technology for heteroatom doping of graphene has been proven to be an effective way to adjust and improve the physical and chemical properties of graphene [77]. Ren et al. [78] used PLD technology to deposit graphene films with different nitrogen doping levels (NG) by adjusting the nitrogen pressure during the deposition process, and the results showed that nitrogen atoms replaced carbon atoms in the graphene lattice in the form of pyridine N and pyrrole N, thus being doped into the graphene film. Meanwhile, through Raman scattering measurements of Rhodamine 6G (R6G) molecules, it was found that nitrogen doping can increase the additional charge carriers and change the electronic structure of graphene films, thereby effectively enhancing the Raman signal of NG films. The relative enhancement factor first increases with the increase in nitrogen doping content and gradually decreases after reaching a maximum of 2.5 times (Figure 4c), which indicates that the relative enhancement factor of Raman spectra can be adjusted by regulating the N content in NG films, thereby promoting graphene-enhanced Raman scattering.

Surprisingly, PLD technology can be used to functionalize and modify graphene 2D film materials, thereby further enhancing and enriching the application value and scope of graphene while regulating its electronic structure and physicochemical properties [93]. Plšek et al. [70] prepared graphene catalysts functionalized with ceria nanoparticles using PLD technology and studied their effect on methanol catalytic conversion, and they found that graphene can effectively stabilize the high reducibility of ceria/graphene catalysts and promote the development of catalytic products towards dehydrogenation.

The use of PLD technology can not only prepare 2D film materials of graphene, doped graphene, and functionalized graphene but also grow reduced graphene oxide (rGO) and ternary mixed graphene, which further enriches the application scope of PLD technology in the preparation of graphene-related 2D film materials [94]. Juvaid et al. [70] deposited wafer-level rGO films using PLD technology and investigated the effects of different deposition conditions on the physicochemical properties of rGO films, and their results showed that adding a small amount of oxygen during PLD deposition was very beneficial for preparing high-quality rGO films, and with the increase in oxygen pressure, the sp^2^ peak of carbon in rGO films gradually decreased, while the sp^3^ peak intensity increased (Figure 4d). At the same time, the rGO film prepared by PLD was an ultrathin p-type transparent conductor (transparency >97%), and the rGO film grown in the presence of 0.5 mTorr oxygen had a resistivity of 30.3 mΩ cm, a carrier concentration of 2.3 × 10^13^ cm^−2^, and a mobility of 5.11 cm^2^V^−1^s^−1^, which exhibits excellent optoelectronic properties. Alshareefi et al. [81] used PLD technology to prepare ternary hybrid nanomaterials of silica nanowires/graphene/zinc oxide nanoparticles (SiO NWs/G/ZnO NPs) and found that, compared to SiO NWs, SiO NWs/G, and SiO2 NWs/ZnO NPs, SiO NWs/G/ZnO films can effectively improve its light reflectivity (its reflectivity is 60% for UV light, the reflectivity in the visible light region is 25%, and the reflectivity at 1000 nm is 100%) (Figure 4e), while the effect on the optical energy gap is insignificant, and the lowest resistivity is 1.13 × 10^−2^ Ω.cm, the highest carrier concentration is 3.92 × 10^18^ cm^−3^, the smallest Hall coefficient is 1.59 cm^3^C^−1^, the optimal conductivity is 8.87 × 10^1^ Ω^−1^ cm^−1^, and significant mobility is 1.41 × 10^2^ cm^2^V^−1^s^−1^. Meanwhile, SiO NWs/G/ZnO films can effectively convert incident photons into current signals, resulting in outstanding photoresponsivity (0.79A/W), specific detectability (35.68 × 10^6^ cm·Hz^1/2^·W^−1^), and quantum efficiency (107.43%), thereby exhibiting excellent optoelectronic properties and becoming a favorable competitor for high-quality photodetectors.

#### 3.1.2. Application of PLD in Diamond-Like Carbon (DLC)

Diamond-like films have attracted widespread attention due to their excellent mechanical, tribological, optical, and biological properties, such as high hardness, light transmittance, low friction coefficient, and extremely strong scratch resistance, which make them have potential applications in fields such as microelectromechanical devices, precision machinery, the healthcare industry, color gradient coatings, biomimetic materials, and energy-saving devices [95]. DLC is a metastable form of carbon that consists of a combination of amorphous and crystalline phases, rather than having a single composition [96]. The atoms in DLC are randomly placed in a continuous random network with variable sp^3^ (diamond-like) and sp^2^ (graphitic) bonds [97]. Therefore, due to the presence of triangular (sp^2^) and tetrahedral (sp^3^)-bonded carbon substrates, DLC exhibits excellent tunable electrical properties, from semiconductor to insulation [98]. In DLC films, each carbon atom can be connected to the other four carbon atoms through covalent bonds and van der Waals forces, forming a complex spatial network of cross-structure [99].

As one of the allotropes of carbon, DLC has both a graphite-like sp^2^ structure and diamond-like sp^3^ structure, which makes it exhibit the properties of both graphite and diamond, such as excellent hardness, tunable electronic properties, outstanding transparency, good insulating properties, etc. These rich physicochemical properties make DLC have a wide range of potential application values and a range of applications [100]. PLD technology has been proven to be one of the most effective methods for the successful preparation of DLC films, which can be prepared with high-purity, oxygen-free, and hydrogen-free DLC films [101]. Valcheva et al. [83] prepared DLC films using PLD technology, and their study showed that DLC films with different structural compositions (i.e., the ratio of sp^3^/sp^2^ hybridized carbon) can be prepared by changing the deposition conditions, and as the thickness of the DLC films increases, the content of sp^3^ hybridized carbon gradually decreases, and the resistivity also decreases. The thinnest DLC film (0.5 nm) exhibits a relatively low specific resistance (1.5 × 10^−3^ Ω·m), which makes the DLC film increasingly exhibit graphene-like properties. Meanwhile, the variation of resistivity/conductivity of DLC films with the temperature was investigated, and it was found that, at temperatures below 300 K, the resistivity of all DLC films decreased with the increasing temperature (Figure 4f), which indicates that their DLC films exhibit non-metallic behavior, which is contrary to the metallic behavior of highly conductive dielectrics such as graphene. Lu et al. [84] used the PLD technique to deposit DLC films on silicon substrates and showed that DLC films prepared under high laser fluence exhibit a high infrared transmittance and refractive index and low extinction coefficient. Meanwhile, the composite film made by inserting a SiC film layer in the middle of two DLC films can improve its adhesion properties without affecting the infrared transmittance, thus enhancing the critical load of the DLC/SiC/DLC composite film, in which the critical load is 218.9 mN, which is 24% higher than that of the single-layer DLC film (Figure 4g). The DLC films deposited on the Si substrate using the PLD technique have good electrochemical properties, especially after pulsed laser annealing [85]; the maximum area-specific capacitance of the DLC film was 48.25 mF/cm^2^, and the capacitance retention and the coulombic efficiency after 5000 cycles were 97.3% and 93.5% (Figure 4h). However, the maximum specific capacitance of the original DLC film was only 37.2 mF/cm^2^, with a capacitance retention rate and coulombic efficiency of 93.7% and 91%, respectively (Figure 4i).

#### 3.1.3. Application of PLD in Carbon Nanotube (Sheet) Films

Carbon nanotube (sheet) films, as a kind of carbon-based 2D material that is easy to functionalize, have the characteristics of high conductivity, high strength, high flexibility, and high transparency and are widely used in important fields such as solar cells and photodetectors [102]. The preparation of carbon nanotube (sheet) films or functionalization modification by PLD technology can effectively control and regulate their physicochemical properties and avoid contamination from chemical reagents [103]. Farman et al. [87] deposited single-walled carbon nanotubes (SWCNTs) and multi-walled carbon nanotubes (MWCNTs) on porous silica glass (PS) substrates by PLD technology and prepared nanocarbon silicon solar cells by combining them with n-type conductive monocrystalline silicon wafers and, at the same time, investigated the effects of different carbon-based films on the conversion efficiency of nanocarbon silicon solar cells. The results showed that both SWCNT/PS films and MWCNT/PS films exhibited good surface morphology and excellent uniformity, with an average surface roughness of 0.39 nm and 1.78 nm and root mean square variances of 0.45 nm and 2.09 nm, respectively. By analyzing the current density–voltage characteristics of carbon-based nano-silicon solar cells, it was found that the SWCNT cells exhibited a higher short-circuit current density (30 mA/cm^2^) and power conversion efficiency (5.6%) compared to graphene cells and MWCNT cells (Figure 4j). Moise et al. [88] used PLD to directly deposit SWCNTs on carbon fibers and found that their average diameter was 1.35 nm, and the intensity ratio (ID/IG) between the D and G peaks in Raman spectroscopy was 0.07, which implies that the SWCNTs have high quality. At the same time, the deposition of SWCNTs on carbon fibers increased their surface roughness and total surface area, resulting in a smaller contact angle with polar liquids, whereas SWCNTs exhibited a higher contact angle for non-polar liquids due to their hydrophobicity and oleophobicity and due to the relatively low temperature of the PLD deposition process, which made the SWCNTs deposited on the carbon fibers maintain their original tensile strength.

### 3.2. Application of PLD in Sulfides

Two-dimensional sulfides have attracted widespread attention due to their excellent performance in the fields of thermodynamics, optics, and electronics [104]. Among them, transition metal disulfides (MoS_2_, WS_2_, etc.) have been widely used in microelectronics, photodetection, optoelectronic sensing, and other fields due to their controllable bandgap, high carrier mobility, and excellent optical absorption coefficient [105]. Meanwhile, other 2D sulfides (NiS_2_, SnS_2_, Cu_2_ZnSnS_4_, etc.) have also shown potential application values in many fields due to their unique properties [106]. PLD, as a technology that can precisely control the stoichiometric ratio, has a significant advantage in the preparation of high-quality 2D sulfides [107]. Table 2 is the summary of the deposition conditions and properties of sulfides by PLD.

#### 3.2.1. Application of PLD in MoS_2_

**Figure 5:**
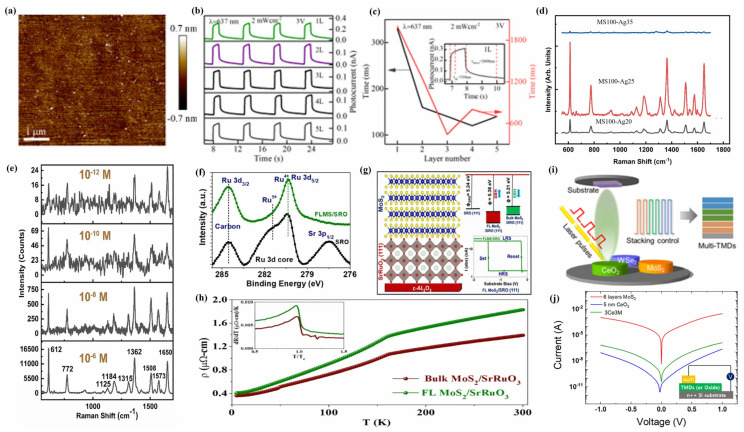
(**a**) The AFM image of a MoS_2_ film on a c-Al_2_O_3_ substrate. (**b**) The time-resolved photocurrent of MoS_2_ films with different layers. (**c**) The layer-dependent rising and decay response times. The inset presents the rising and decay response times of the monolayer MoS_2_ film. Reproduced from Ref. [108], Copyright 2021, Journal of Infrared and Millimeter Waves. (**d**) The SERS spectra of Rhodamine 6G at a concentration of 10^−6^ M on the samples. (**e**) The SERS spectra of R6G with a decreasing concentration on the MS100-Ag25 sample. Reproduced from Ref. [112], Copyright 2022, Elsevier. (**f**) Shift in XPS spectra of SrRuO_3_/c-Al_2_O_3_ and FL MoS_2_/SrRuO_3_/c-Al_2_O_3_. (**g**) Temperature-dependent resistivity data for bulk and monolayer MoS_2_ on SrRuO_3_(111) confirms a higher resistance for the FL MoS_2_ film over BL MoS_2_. (**h**) Structure and calculated work functions of MoS_2_/SrRuO_3_/c-Al_2_O_3_ heterojunction films. Reproduced from Ref. [113], Copyright 2023, American Chemical Society. (**i**) Schematic of a sequential PLD system for the growth of MHJ-TMDs. (**j**) I–V curve for two-terminal tunneling devices based on 6 layers of MoS_2_, 5 nm CeO_2_, and 3Ce3M. All samples were fabricated on heavily doped n-type silicon (inset schematic). Reproduced from Ref. [115], Copyright 2021, American Chemical Society.

MoS_2_, as a member of the 2D transition metal disulfide family, consists of S-Mo-S atomic layers with strong covalent bonds within each layer and weak van der Waals forces between layers [128]. Meanwhile, MoS_2_ exhibits anisotropy due to its equal crystal structures of trigonal (1T), rhombohedral (3R), and hexagonal (2H), which makes it outstanding in devices such as photodetectors, film transistors, supercapacitors, and photocatalysts [129]. Zhang et al. [108] prepared pure 2H-phase polycrystalline MoS_2_ films with a controllable film thickness on c-Al_2_O_3_ substrate using the PLD technique, which have extremely high flatness (roughness of 0.162 nm) (Figure 5a) and good thickness uniformity, with a grain size of 25.3 nm and a bandgap of 1.86 eV. Meanwhile, analysis of the photoelectric properties of MoS_2_ films with different thicknesses revealed that single-layer MoS_2_ films exhibit the strongest photocurrent (0.32 nA) and photoresponse (3 mAW^−1^) (Figure 5b), but their response to rise and decay time is the slowest (Figure 5c), which is attributed to the fact that the increase in the number of MoS_2_ film layers alters their bandgap structure, resulting in a shorter lifetime and response time of photogenerated carriers.

PLD technology can be used for doping and the heterostructure modification of MoS_2_, thereby effectively controlling and altering its electronic structure, optical properties, and electrical characteristics [130]. Adding alkali metals or alkaline earth metals with smaller ionic radii to MoS_2_ can promote the phase transition of MoS_2_ films and change their physical properties, thus gaining wide applications [131]. Hrda et al. [111] used PLD technology to investigate the effect of lithium doping on the electronic transport characteristics of MoS_2_ films, and the results showed that lithium doping did not change the crystal structure and semiconductor properties of MoS_2_ films but could affect their transport characteristics. Whether it is a pure MoS_2_ film or lithium-doped MoS_2_ film, their electron migration is an insulation transfer. Among them, the disorder of pure MoS_2_ film is caused by grain boundaries, and with the increase in Li doping amount in MoS_2_ film, its resistance and activation energy rapidly increase, which means that lithium doping introduces additional disorder.

At the same time, it was also found that the combination of MoS_2_ films with finely controllable layers and plasma metal nanomaterials can significantly enhance the Raman spectral signal, thereby improving the detection range and limit of Raman spectroscopy [132]. The MoS_2_ film doped with silver nanoparticles (Ag-MoS_2_) prepared using PLD technology exhibits higher surface-enhanced Raman scattering (SERS) activity towards R6G than pure MoS_2_ film and pure silver nanoparticles (Ag NPs) [112], which may be the result of the increased surface roughness, electromagnetic enhancement, and chemical enhancement of the MoS_2_ film itself caused by Ag-MoS_2_ film. As the concentration of silver nitrate precursor increases, the content of Ag NPs in the MoS_2_ film also gradually increases, resulting in an increase in the SERS activity of the R6G by the Ag-MoS_2_ film, with a maximum enhancement factor of 1.8 × 10^8^. However, when the concentration of silver nitrate precursor continues to increase to 35 mmol, Ag NPs aggregate in the MoS_2_ film, which inhibits the interaction between the MoS_2_ film and the R6G, thereby reducing its SERS activity towards R6G (Figure 5d). Meanwhile, the Ag-MoS_2_ film can achieve a minimum detection limit of 10^−12^ M for the R6G (Figure 5e). It is interesting that, after 30 min of UV irradiation, the SERS intensity of the R6G on the Ag-MoS_2_ film is twice that of the nonirradiated sample, and this process is reversible. When the UV irradiation is turned off, its SERS intensity can return to the same level as the nonirradiated sample.

The heterojunction between 2D MoS_2_ and transition metal oxides can be achieved through PLD technology, forming a 2D heterojunction material with a 3D structure and exhibiting unique electronic structures and novel physical phenomena at the 2D/3D interface [133]. Parmar et al. [113] prepared MoS_2_/SrRuO_3_/c-Al_2_O_3_ heterojunction films using PLD technology and studied their structural properties, and they found that Ru and S can form chemical hybridization in the MoS_2_/SrRuO_3_/c-Al_2_O_3_ heterojunction films, which strongly affects the electronic properties of MoS_2_ (Figure 5f). At the same time, the resistivity of the few-layer (FL) MoS_2_/SrRuO_3_ heterojunction film (1.83 μΩ cm) is higher than that of the bulk layer (BL) MoS_2_/SrRuO_3_ heterojunction film (1.39 μΩ cm), and a ferromagnetic transition occurs at a temperature of 160 K, indicating that the heterojunction changes the spin–spin correlation near the critical temperature (Figure 5g). In addition, as the amount of MoS_2_ in the MoS_2_/SrRuO_3_/c-Al_2_O_3_ heterojunction increases, its work function and Schottky barrier height also show significant differences (Figure 5h). The strong Ru-S bond hybridization, lower work function, and controllable Schottky barrier height in MoS_2_/SrRuO_3_/c-Al_2_O_3_ heterojunction films are the reasons for the differences in device characteristics of variable resistance memory, while the few-layer MoS_2_ resistance switch is the result of the combined effects of the SrRuO_3_/MoS_2_ Schottky junction, sulfur vacancies, Ru-S hybridization, and the polymorphism of MoS_2_ in the heterojunction.

In addition, PLD technology can also enable MoS_2_ to form complex multiheterojunction (MHJ) structures, exhibiting novel physical and chemical properties [134]. The synergistic effect of MoS_2_ and oxide materials in forming heterojunctions can improve the sensing performance, electrocatalytic performance, and electron mobility of MoS_2_ devices [135]. Seo et al. [115] used PLD technology to uniformly grow MHJ-TMD composite films with different stacking orders and numbers of layers on SiO_2_ substrates by controlling the multi-axis target rotation system and modulating the number of laser pulses (Figure 5i). Due to the lattice mismatch between the WSe_2_ layer and the MoS_2_ layer in the MHJ-TMD composite films, significant lattice strain and dielectric shielding occurred between them, which led to a good interlayer coupling effect between each layer of the MHJ-TMD composite films. At the same time, the electrical properties of MHJ-TMD films grown by PLD were measured, and it was found that the six-layer MoS_2_ films exhibited a higher tunneling current than 5 nm CeO_2_, while the current density of the 3 nm CeO_2_/tri-layer MoS_2_ (3Ce3M) film was between that of the six-layer MoS_2_ and 5nm CeO_2_ (Figure 5j), which implies that the tunneling current of MHJ-TMD composite films can be effectively controlled by adjusting the oxide layer thickness and composite structure.

#### 3.2.2. Application of PLD in WS_2_

**Figure 6:**
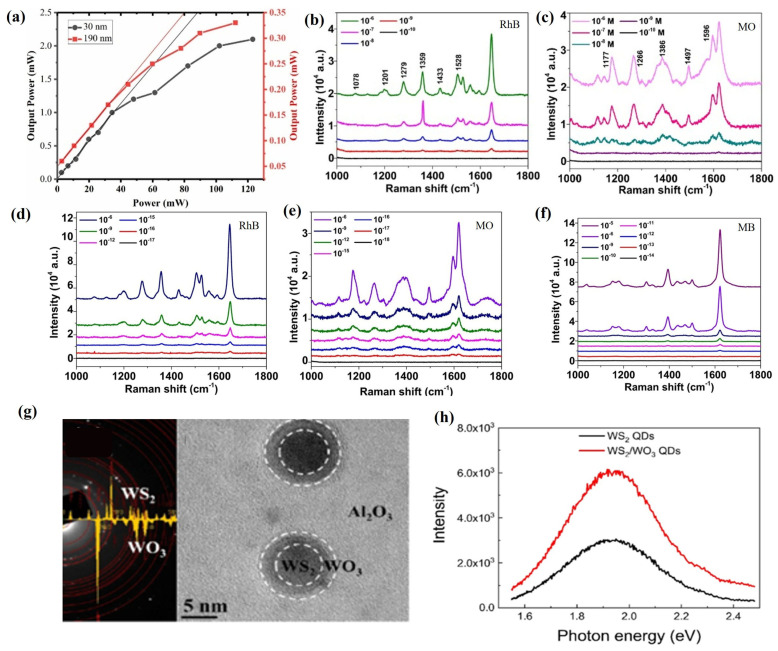
(**a**) Optical limiting of WS_2_ films. Reproduced from Ref. [118], Copyright 2024, Elsevier. (**b**) RhB concentration-dependent SERS spectra as measured on a bare WS_2_ SERS substrate. (**c**) MO concentration-dependent SERS spectra as measured on a bare WS_2_ SERS substrate. (**d**) RhB concentration-dependent SERS spectra as measured on a bare Ag-WS_2_ SERS substrate. (**e**) MO concentration-dependent SERS spectra as measured on a bare Ag-WS_2_ SERS substrate. (**f**) Concentration-dependent SERS study of MB dye with a 633 nm wavelength laser. Reproduced from Ref. [120], Copyright 2024, Elsevier. (**g**) Electron diffraction pattern and planar HRTEM image of a WS_2_/WO_3_ heterostructure QDs in an amorphous Al_2_O_3_ matrix. (**h**) PL spectra taken from the WS_2_/WO_3_ heterostructure QDs (red) and WS_2_ QDs (black) measured under the excitation of a 514 nm laser wavelength. Reproduced from Ref. [121], Copyright 2020, Elsevier.

WS_2_, as another typical transition metal disulfide, has excellent chemical, optical, and electronic properties such as strong thermal stability, adjustable bandgap, and excellent spin-orbit coupling and has been widely used in a variety of fields such as sensors, energy storage devices, photocatalysis, solar cells, etc. [118]. Khanzadeh et al. [118] used the PLD technique to grow WS films with a hexagonal structure on a glass substrate, where WS_2_ films with a thickness of 30 nm showed a direct bandgap of 1.98 eV and a defect energy of 1.28 eV, while WS_2_ films with a thickness of 190 nm exhibited an indirect bandgap of 1.41 eV and a defect energy of 0.83 eV. Meanwhile, compared to the 190 nm thick WS_2_ film, the WS_2_ film with a thickness of 30 nm exhibits more effective optical limiting behavior (Figure 6a).

Like MoS_2_, WS_2_ films can also serve as substrates for SERS to improve the accuracy, sensitivity, and efficiency of Raman spectroscopy [136]. Kaushik et al. [120] deposited WS_2_ films on Si substrates using PLD technology and used them as SERS substrates to detect low concentrations of environmental pollutants (Rhodamine B (RhB) and methyl orange (MO)). The results showed that the detection limit of WS_2_ films prepared by PLD as SERS substrates for RhB and MO dyes could reach 10^−9^ M, with Raman enhancement factors of 2.4 × 10^7^ and 8.5 × 10^6^, respectively (Figure 6b,c). Meanwhile, the use of Ag nanoparticles to modify WS_2_ films can significantly enhance the electromagnetic effect of Ag-WS_2_ composite films, thereby improving their SERS performance towards organic dyes. The detection limits of the Ag-WS_2_ composite SERS substrate for RhB and MO dyes are 10^−16^ M and 10^−17^ M, respectively, with Raman enhancement factor distributions of 1.0 × 10^8^ and 2.6 × 10^7^ (Figure 6d,e). Under different wavelengths of the laser, the detection limit of methylene blue (MB) on the Ag-WS_2_ composite SERS substrate can reach 10^−13^ M, with an enhancement factor of 5.8 × 10^7^ (Figure 6f). In addition, the Ag-WS_2_ composite SERS substrate exhibits excellent reproducibility and good stability in the detection of organic dyes.

In addition, the use of PLD technology to prepare WS_2_ heterojunction structures is crucial for improving their optoelectronic properties [137]. The use of PLD technology and rapid thermal annealing technology can successfully prepare WS_2_/WO_3_ heterostructure quantum dots composed of hexagonal WS_2_ and monoclinic WO_3_ structures (Figure 6g) [121]. At the same time, photoluminescence (PL) detection was performed on WS_2_/WO_3_ heterojunction quantum dots at a laser wavelength of 514 nm, and it was found that WO_3_ in the WS_2_/WO_3_ heterojunction could effectively suppress the surface defects of WS_2_ quantum dots, thereby significantly improving the PL intensity of WS_2_/WO_3_ heterojunction quantum dots (Figure 6h). In addition, time-resolved photoluminescence (TRPL) spectroscopy measurements were performed on WS_2_/WO_3_ heterostructure quantum dots and WS_2_ quantum dots. It was found that the fast decay time of WS_2_/WO_3_ heterostructure quantum dots was 1.0 μs and the slow decay time was 4.1 μs, while the fast decay time and slow decay time of WS_2_ quantum dots were only 0.8 μs and 3.8 μs, respectively, which indicates that WS_2_/WO_3_ heterostructure quantum dots have a longer PL lifetime than WS quantum dots and enhance their photoluminescence quantum yield.

#### 3.2.3. Application of PLD in Other Sulfides

**Figure 7:**
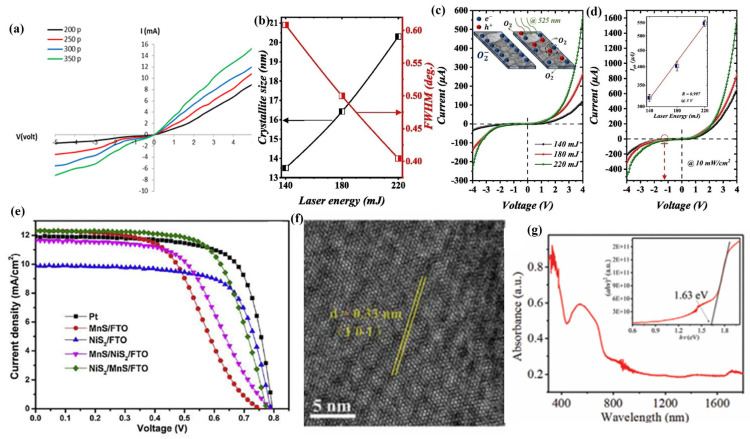
(**a**) I–V characteristic of the SnS/Si heterojunction at present light. Reproduced from Ref. [122], Copyright 2023, International Journal of Nanoelectronics and Materials. (**b**) In-depth CdS film XRD parameters concerning the calculated crystallite size according to the Debye Scherrer formula and FWHM as a function of laser energy. (**c**) Profile of laser energy influence on the fabricated CdS photodetector; I–V characteristics under dark conditions. (**d**) Profile of laser energy influence on the fabricated CdS photodetector; I–V characteristics under light conditions. Reproduced from Ref. [123], Copyright 2023, Elsevier. (**e**) Photocurrent density–voltage curves of Pt, NiS_2_, MnS, NiS_2_/MnS, and MnS/NiS_2_. Reproduced from Ref. [124], Copyright 2021, Elsevier. (**f**) HRTEM image of the PLD-derived ZIS. (**g**) UV–Vis absorption spectrum of a ZIS nanofilm. Inset: The corresponding Kubelka–Munk transformation. Reproduced from Ref. [125], Copyright 2022, John Wiley and Sons.

In addition to the preparation of transition metal disulfides, PLD has also been widely used in the preparation of other 2D sulfides such as SnS, CdS, Cu_2_ZnSnS_4_ (CZTS), and ZnIn_2_S_4_ (ZIS) [138]. Kadhim [122] used PLD technology to deposit SnS films with high crystallinity and an orthogonal crystal structure, and with the increase in laser pulses, their thickness, grain size, average diameter, and roughness all increased. Meanwhile, with the increase in laser pulses, the absorption coefficient of the SnS film gradually increases, while the transmittance and direct bandgap energy (E_g_) gradually decrease. In addition, SnS films exhibit an n-type semiconductor type, where the charge carrier concentration increases with the increase in laser pulses, while the carrier mobility decreases with the increase in laser pulses. After analyzing the photosensitivity of SnS film devices, it was found that, with the increase in laser pulses, the photocurrent of SnS film devices gradually increased (Figure 7a), while the capacitance gradually decreased, and their maximum spectral sensitivity, specific detectability, and quantum efficiency were 0.72 A/W, 2.85 × 10^11^ cm·Hz^1/2^·W^−1^, and 70.18%, respectively.

Meanwhile, the preparation of cadmium sulfide (CdS) films with excellent photovoltaic properties by PLD technology has also attracted attention [139]. Aldaghri et al. [123] used the PLD technique to prepare CdS films with a hexagonal wurtzite structure, and by controlling the laser energy during the deposition process, the grain size, crystallinity, surface morphology, and optical bandgap of the CdS films can be effectively controlled (Figure 7b). Meanwhile, the dark/light I–V curve, light–dark current ratio, photoresponsivity, external quantum efficiency, and specific detectivity of CdS photodetectors all showed a positive correlation with laser energy (Figure 7c,d). In addition, the quality factors (photoresponsivity, external quantum efficiency, and specific detection rate) of CdS photodetectors are negatively correlated with the applied illumination light power, and the CdS photodetectors prepared under the condition of laser energy of 220 mJ exhibited the highest external quantum efficiency value (129.4%) and the lowest response time (179 ms) and recovery time (161 ms).

In addition, the preparation of 2D sulfide films by PLD technology as a counter electrode (CE) for dye-sensitized solar cells (DSSCs) to replace the precious metal counter electrode, such as platinum, has attracted attention [140]. Cao et al. [124] used PLD technology to prepare NiS_2_/MnS/FTO and MnS/NiS_2_/FTO heterojunction films with good crystallization properties and high purity, in which the NiS_2_/MnS/FTO films have higher surface roughness and a specific surface area, which is very favorable to improve the electrocatalytic activity of CEs, because it can provide more electrocatalytic activity spots. Meanwhile, the NiS_2_/MnS/FTO heterojunction film also exhibited excellent photovoltaic properties and electrocatalytic performance, and its power conversion efficiency (PCE) could reach 6.44%, which was very close to the power conversion efficiency (6.84%) of the Pt counter electrode (Figure 7e).

It is gratifying that PLD technology has also demonstrated unique advantages in the preparation of multicomponent 2D sulfide films [141]. Ye et al. [125] successfully prepared a high-crystallinity polycrystalline ZnIn_2_S_4_ (ZIS) nanofilm with a hexagonal crystal structure using PLD technology, which had a crystal plane spacing of 3.3 Å in the (101) lattice plane (Figure 7f). Meanwhile, the ZIS nanofilm has strong light absorption properties and photosensitivity with a band gap of 1.63 eV (Figure 7g). The response time and recovery time of the ZIS photodetector were 15.34 ms and 14.43 ms, respectively, and its photocurrent increased with the increase in power density, whereas the responsivity, external quantum efficiency, and detection rate decreased with the increase in power density, and its optimal responsivity, external quantum efficiency, and detection rate were 1.4 AW, 430%, and 9.8 × 10^9^ Jones (1 Jones = 1 cm Hz^1/2^ W^−1^). In addition, the ZIS photodetector exhibits a strong photoresponse in both the UV-to-visible light ranges, and it shows good stability over 1900 photoswitching cycles and 1 month of storage in air under unencapsulated conditions. Esterrich et al. [126] successfully prepared Cu_2_ZnSnS_4_ (CZTS) films using PLD technology, and they found that the composition of CZTS films changed continuously with different deposition temperatures. When the deposition temperature was 450 °C, CZTS thin films had good stoichiometry and crystallinity, but when the temperature increased to 500 °C, CZTS thin films underwent substantial decomposition, and their Sn completely disappeared.

### 3.3. Application of PLD in Oxides

Two-dimensional metal oxide film materials have been widely used in many fields, such as photocatalysis, electrocatalysis, sensors, supercapacitors, solar cells, photodetectors, and film transistors, due to their diversity of natural constituent elements, adjustable photovoltaic properties, excellent electrochemical properties, outstanding thermal stability, and unique electronic structure and electromagnetic properties, which has led to an extensive research boom [142]. Among the many methods for preparing 2D metal oxide film materials, PLD technology has shown unique advantages in terms of film preparation efficiency, parameter and structure adjustability, environmental friendliness, and film purity, thus becoming the preferred method for preparing high-quality 2D metal oxide film materials [143]. Table 3 is the summary of the deposition conditions and properties of oxides by PLD.

In the process of preparing 2D metal oxide film materials by PLD technology, the electronic structure and physicochemical properties can be effectively changed by adjusting the deposition parameters so as to prepare high-quality 2D film materials with different characteristics [162]. Hadi et al. [144] used PLD technology to prepare ZnO films with a hexagonal wurtzite-phase polycrystalline structure by changing the laser energy density. As the pulse laser energy increased, the surface roughness, thickness, and grain size of the ZnO film increased, while its bandgap energy gradually decreased. Meanwhile, with the increase in laser energy, the saturation current density of ZnO film devices increases, while the ideality factor and barrier heights gradually decrease. In addition, analysis of the optoelectronic properties of ZnO film devices revealed that they exhibit a significant spectral response in the wavelength range from ultraviolet to near-infrared light, and with the increase in laser energy, the spectral responsivity, specific detection rate, and external quantum efficiency (EQE) of ZnO film devices gradually increased. The ZnO film devices deposited at 800 mJ laser energy showed the highest spectral responsivity (0.46 A/W), specific detection rate (29.48 × 10^10^ Jones (cm·Hz^1/2^W^−1^)), and external quantum efficiency (92.31%). Biswa et al. [151] used PLD technology to prepare polycrystalline rutile-phase SnO_2_ films with different morphologies at different deposition temperatures. As the deposition temperature increased, the crystallinity and grain size of the SnO_2_ films also increased. The lattice stripe spacing of the SnO_2_ films prepared at 400 °C along the (110), (020), and (011) planes was 0.342 nm, 0.236 nm, and 0.269 nm, respectively. At the same time, electrochemical analysis was conducted using SnO_2_ film as the anode of sodium ion batteries, and it was found that the SnO_2_ film prepared at 300 °C exhibited excellent discharge capacity, stable cycling performance, and good coulombic efficiency. After 70 cycles at a current density of 20 mA/g, it could still maintain a discharge capacity of 424 mAh/g, and the coulombic efficiency after 200 cycles at a current density of 130 mA/g was as high as 96%.

**Figure 8:**
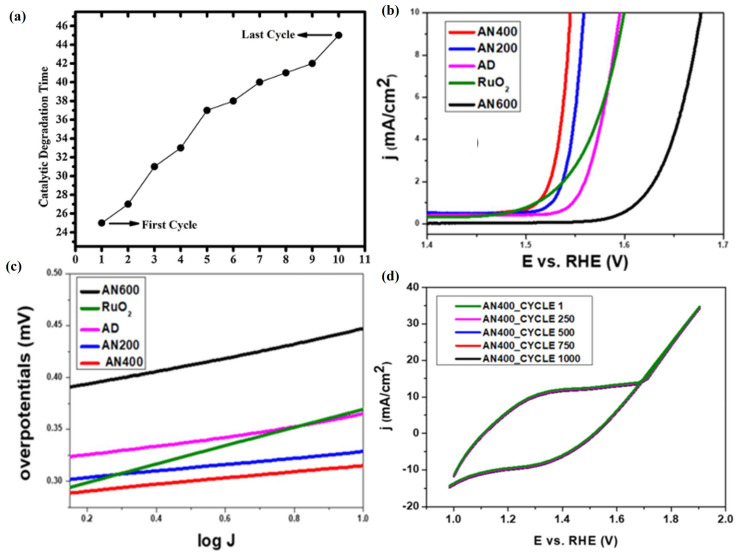
(**a**) Stability of Ag-doped CuO film for the degradation of 4-nirophenol to 4-aminophenol. Reproduced from Ref. [155], Copyright 2020, Elsevier. (**b**) I–R-compensated LSV curves for as-deposited, annealed samples and RuO_2_. (**c**) Tafel plots for as-deposited, annealed samples and RuO_2_. (**d**) Recyclability test by performing cyclic voltammetries on AN400 coating for 1000 cycles. Reproduced from Ref. [160], Copyright 2022, Springer.

Meanwhile, due to the easy controllability of PLD technology, its structure and properties can be controlled through element doping during the preparation of 2D metal oxide film materials, thereby enriching and enhancing its application scope and value [163]. Gupta et al. [147] used PLD technology to deposit ZnO films (NZO) with different Ni doping levels (0%, 3%, 5%, and 7%), and the results showed that, as the Ni doping level increased, the effective atomic mass and lattice parameters in the NZO film changed, thereby affecting its lattice vibration frequency and orderliness, resulting in an increase in the force constant of the Zn-O bond in the NZO film, a decrease in bond length, and a gradual decrease in grain size and average roughness. Meanwhile, the NZO film exhibited significant transmittance in the UV–Visible region (300–800 nm), but with the doping of Ni in the ZnO film, the transparency and bandgap energy of the NZO film gradually decreased. When the Ni doping content reached 7%, the bandgap value of the NZO film was 3.11 eV. In addition, NZO films exhibit significant photoluminescence emission in the visible light region at 430nm, and with the increase in Ni doping in ZnO, the energy of E_2_ high modes in the Raman spectra of NZO films undergoes a red shift. Menazea et al. [155] deposited CuO films doped with silver nanoparticles using PLD technology and investigated their catalytic degradation performance of 4-nitrophenol, and the results showed that the doping of Ag NPs on CuO films improved the crystallinity of Ag/CuO films and increased their grain size (98.45 nm), average roughness (47 nm), and visible light transmittance (97%) while reducing their bandgap value (2.43 eV). In addition, Ag/CuO film can complete the catalytic degradation of 4-nitrophenol in 25 min, while CuO film takes 60 min to complete the catalytic degradation. After using Ag/CuO film for the cyclic catalytic degradation of 4-nitrophenol 10 times, its catalytic degradation time is still lower than that of CuO film (Figure 8a), which means that Ag/CuO film can significantly improve the degradation efficiency of 4-nitrophenol and has good catalytic stability. Enad et al. [159] deposited Nd_2_O_3_-doped CuO films (NCO) by using the PLD technique and investigated the effects of different Nd_2_O_3_ doping amounts (1 wt.%, 3 wt.%, and 5 wt.%) on the structure and properties of CuO films, and their results showed that doping Nd_2_O_3_ in monoclinic-structured CuO films can cause a localized shrinkage strain in the lattice of CuO, and the intensity of the Nd_2_O_3_ diffraction peaks was gradually enhanced with the increasing amount of Nd_2_O_3_ doping, while the grain size of the NCO films decreased from 19 nm to 14.7 nm. Meanwhile, the doping of Nd_2_O_3_ in CuO films changed the surface morphology of NCO films and increased their oxygen vacancies, thereby enhancing the interaction between NCO films and gas molecules, which was crucial for improving their gas sensitivity. In addition, the doping of Nd_2_O_3_ in CuO films effectively improves the sensitivity of NCO film gas sensors to NH_3_, and its sensitivity to NH_3_ is gradually enhanced with the increase in Nd_2_O_3_. When the doping amount of Nd_2_O_3_ in NCO films is 5 wt.% at the operating temperature of 50 °C, the NCO film gas sensor exhibits the highest sensitivity of 180% to ammonia gas with a concentration of 79 ppm, which was four times that of pure CuO gas sensors.

As an efficient technique that can effectively maintain the stoichiometric ratio, PLD has an irreplaceable favorable position in the preparation of complex 2D metal oxide film materials and specific heterojunction structure materials [164]. Popat et al. [160] prepared Co-Fe-B-O mixed metal oxide films (CFBO) with partial crystallization using PLD technology, and the CFBO films grown at an annealing temperature of 400 °C exhibited excellent electrochemical activity, with an overpotential at a current density of 10 mA/cm^2^ of 315 mV (Figure 8b), and the Tafel slope was 31.5 mV/dec (Figure 8c). Meanwhile, the presence of B and Fe plays an extremely important role in the generation and regeneration of Co active sites in CFBO films, making their abundant catalytic active sites a decisive factor for the enhancement of electrochemical activity. In addition, the current density of the CFBO films was almost unchanged after 12 h of continuous operation or 1000 cyclic voltammetry scans under 1.55 V vs. RHE conditions (Figure 8d), which implies that the CFBO films prepared by PLD have good stability and recyclability.

### 3.4. Application of PLD in Nitrides

**Figure 9:**
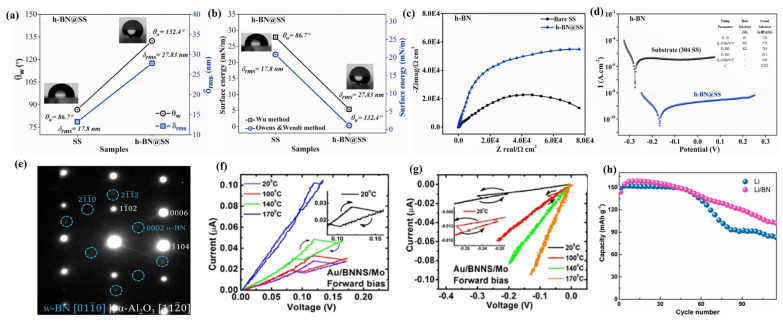
(**a**) Contact angle and surface roughness variation uncoated, and (**b**) surface energy variation for uncoated and h-BN-coated samples. (**c**) Nyquist diagrams. (**d**) Potential vs. current density (Tafel curves) of uncoated and h-BN-coated 304 SS. Reproduced from Ref. [169], Copyright 2021, Elsevier. (**e**) Electron diffraction pattern showing the epitaxial relationship between sapphire and w-BN. Reproduced from Ref. [171], Copyright 2018, Elsevier. (**f**,**g**) Au/BNNS/Mo Schottky contacts with and without the hysteresis effect taken from 0 to ±3 V insteps of 0.01 V in the I–V curve. Reproduced from Ref. [173], Copyright 2021, IOP Publishing Ltd. (**h**) Cycling performances of LFP ll Li and LFP ll Li/BN full cells at a current rate of 0.2C. Reproduced from Ref. [174], Copyright 2021, American Chemical Society.

As a 2D layered compound with excellent chemical and thermal stability, boron nitride exhibits a variety of allotropes with different crystalline phases due to its anisotropy [165]. Among them, hexagonal boron nitride (h-BN), which belongs to the hexagonal crystal system, is called “white graphene”, because it forms a honeycomb structure with alternating arrangements of B and N atoms by relying on the covalent bonds between B and N atoms within the layers and weak van der Waals forces between the layers [166]. At the same time, h-BN 2D films also exhibit unique physicochemical properties, such as an ultra-broad bandgap, low dielectric constant, high thermal conductivity, outstanding band-edge absorption coefficient, and atomically smooth surfaces (without dangling bonds and charge traps), which are widely used in microelectronics, optoelectronic detectors, high-temperature corrosion-resistant and antioxidation protective coatings, etc. [167]. The traditional CVD method can deposit h-BN films on various substrates, but this method has non-negligible problems such as high energy consumption, limited film thickness, high toxicity of the reactive precursor, impurity contamination, and mechanical damage, which affect the performance and application value of h-BN film devices [168]. PLD, as a high-energy and thermally nonequilibrium film growth technique, has become the darling of the current research in the preparation of h-BN films due to its advantages of maintaining excellent stoichiometry and easy control of structural properties [169]. Kumar et al. [169] used PLD technology to grow high-quality h-BN films with uniform distribution on a stainless steel (SS 304) substrate according to the stoichiometric ratios. The deposition of h-BN film changed the wetting characteristics of the SS 304 substrate, transforming it from hydrophilic (contact angle = 86.7°) to hydrophobic (contact angle = 132.4°), which is beneficial for its corrosion resistance (Figure 9a). Meanwhile, the SS 304 deposited with h-BN films exhibited a lower surface energy, which implies that the h-BN films have strong adhesion on the SS 304 (Figure 9b). In addition, the SS 304 with h-BN films deposited by PLD also exhibited a lower current density (13.2 nA/cm^2^) (Figure 9d), lower corrosion rate (11.7 × 10^−3^ mm/y), and higher corrosion resistance (6.28 × 10^6^ Ω cm^2^) (Figure 9c), which suggests that the h-BN films have excellent anticorrosion properties, thus becoming a strong competitor for anticorrosion materials. During the PLD deposition process, the interaction between thermodynamics and the high kinetics of the atoms gives them the ability to overcome the substrate diffusion energy barrier, resulting in the growth of ordered h-BN nanosheets with a lattice spacing of 0.33 nm [167]. Meanwhile, the h-BN films exhibited strong hydrophobicity (contact angle increased from 55° to 60°) and a low friction coefficient (0.0002), which implies that the h-BN films have good lubricity. In addition, the h-BN films exhibit low refractive indices (1.53–1.55) and room temperature single-photon emission in the visible-to-near-infrared spectral range, which is highly advantageous for the design optimization of photonic devices in this spectral range.

As another allotrope of BN films, the wurtzite-phase BN film (w-BN) with a tetrahedral structure also exhibits unique attraction due to its high hardness [170]. Vishal et al. [171] used PLD technology to prepare epitaxial w-BN films with the desired thickness under relatively slow kinetic conditions by adjusting the deposition parameters (Figure 9e). Meanwhile, the phase transition from h-BN to w-BN occurred at the interface of the c-plane sapphire substrate under PLD deposition conditions, due to selective and stronger chemical interactions between O and B atoms in h-BN. In addition, the prepared w-BN films were tested by nanoindentation and found to have a hardness and elastic modulus of 37 GPa and 339 GPa, respectively, indicating their great potential in the microelectromechanical industry.

In addition, PLD technology can also be used to modify h-BN, thereby adjusting the physicochemical properties of h-BN films and promoting the improvement of the device performance [172]. Ortiz et al. [173] deposited carbon-doped h-BN films (BNNS) using PLD technology and found that carbon doping can change the electronic structure and bandgap value of BNNS films, so that they exhibit stable hysteresis properties in a Schottky barrier structure. The doping of carbon in BNNS films not only reduces its bandgap value but also increases boron vacancies, thereby improving its electrical performance at the metal–BNNS–metal interface. In the Au/BNNS/Mo Schottky barrier structure, charge traps are formed under forward bias and have stable hysteresis characteristics over a wide temperature range (20–170 °C) (Figure 9f), while hysteresis is shown to be less stable under reverse bias (Figure 9g). Li et al. [174] used PLD technology to deposit h-BN on Li particle substrates and investigated its effect on the performance of Li batteries, and their results showed that coating h-BN film on the surface of the Li anode can inhibit the degradation of the electrolyte on the Li electrode and promote the uniform deposition of Li, thereby effectively improving the constant current cycling life of lithium metal batteries (more than 1800 h) and reducing their electrochemical impedance. At the same time, it was found before and after the constant current cyclic electrochemical reaction that the Li electrode deposited with the h-BN film did not have any traces of Li metal in the electrolyte, which implies that the h-BN film has excellent mechanical stability and electrochemical inertness. In addition, compared to commercial Li batteries equipped with LiFePO4 cathodes, Li batteries deposited with h-BN have a more outstanding cycling performance, which still maintains 80% of the battery capacity after 80 cycles (Figure 9h). Table 4 is the summary of the deposition conditions and properties of h-BN by PLD.

### 3.5. Application of PLD in Perovskite Materials

**Figure 10:**
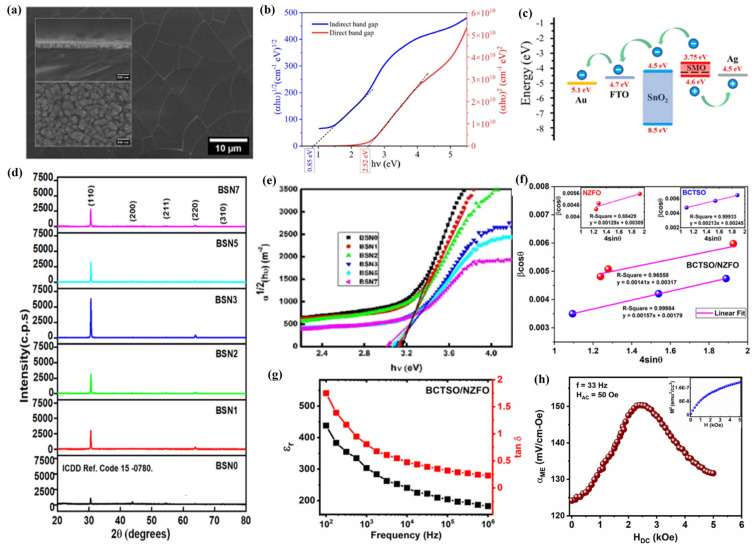
(**a**) FESEM image of SrMnO_3_. The inset shows a cross-sectional view (top) and high magnification surface morphology (bottom). (**b**) UV–Visible spectroscopy of SrMnO_3_ film on quartz. (**c**) Schematic band positioning and band alignment of a SMO perovskite solar cell. Reproduced from Ref. [181], Copyright 2025, Elsevier. (**d**) XRD patterns of BaSnO_3_ films with different Ni doping levels. (**e**) Tauc plot for Ni-doped BaSnO_3_ films. Reproduced from Ref. [188], Copyright 2024, Elsevier. (**f**) Williamson–Hall plots of BCTSO, NZFO, and bilayer BCTSO/NZFO thin films. (**g**) Room temperature frequency dependence of ε_r_ and tanδ of BCTSO/NZFO films. (**h**) Variation of the ME voltage coefficient with the DC magnetic field of the BCTSO/NZFO bilayer composite film, and the inset figure shows the variation of M^2^ with the magnetic field. Reproduced from Ref. [190], Copyright 2025, Elsevier.

Two-dimensional perovskite materials have made significant progress in the fields of optoelectronics, supercapacitors, spintronics devices, sensors, and photodetectors due to their rich element combinations, adjustable bandgap performance, excellent light absorption ability, outstanding carrier mobility, strong thermal stability, outstanding electromagnetic properties, and easily expandable designs for material preparation [177]. Among various preparation methods, PLD technology, as a kind of physical vapor phase method, does not involve solvent reactions in the preparation of 2D perovskite materials, thus avoiding the influence of chemical solvents on material composition and properties under environmentally friendly conditions [178]. Meanwhile, PLD technology can also achieve efficient design of atomic layer precision, thus ensuring precise control of the properties of 2D perovskite materials [179]. In addition, PLD technology has unique advantages in maintaining material stoichiometry and plays an irreplaceable role in ensuring the chemical purity and compositional consistency of 2D perovskite materials [180]. Vikas et al. [181] used PLD technology to prepare SrMnO_3_ (SMO) films as a cubic symmetric structure with an excellent stoichiometry ratio (Sr/Mn~1.0), and its root mean square (RMS) roughness is 5.69 nm (Figure 10a), which is favorable to improve the hole mobility. Meanwhile, oxygen vacancies formed by strain and non-stoichiometry were also discovered in SMO films, with direct and indirect band gaps of 2.52 eV and 0.85 eV, respectively (Figure 10b). In addition, Hall measurements of the SMO films revealed a conductivity of 5.56 × 10^6^ S/cm, a carrier density of 1.37 × 10^12^ cm^−3^, and a mobility of 24.5 cm^2^/V·s at room temperature, and ultraviolet photoelectron spectroscopy (UPS) analysis of the SMO films revealed that the conduction band minima (CBMs), work function, and valence band maxima (VBMs) of the SMO films were 3.75 eV, 4.28 eV, and 4.6 eV, respectively (Figure 10c), which lays a solid foundation for the further construction of photovoltaic devices.

The epitaxial growth of 2D perovskite materials and the formation of heterojunction structures by PLD technology are highly effective in improving and changing their physicochemical properties [182]. Cui et al. [62] used PLD technology to incommensurately epitaxially grow cubic crystal-structured CsPbBr_3_ (100) perovskite films on Si (100) substrates, which exhibit a good stoichiometric, uniform grain size (root mean square roughness of 5.36 nm), stable epitaxial layers, a precisely controllable Si/CsPbBr_3_ interface, and excellent optical properties. Meanwhile, CsPbBr_3_ perovskite film photodetectors exhibit a distinct type II p-n heterojunction structure and good cycling and air stability, with a response/recovery time of 4.2/6.5 ms, and the on/off ratio is significantly improved with the increase of bias under laser irradiation at a wavelength of 650 nm. Under laser irradiation at a wavelength of 520 nm, the photogenerated carriers of CsPbBr_3_ perovskite thin film photodetectors undergo a tunneling effect between silicon and CsPbBr_3_ perovskite films, which led to a responsivity of 780 mA/W and a specific detection ratio of 6.78 × 10^11^ Jones, as well as significantly enhanced external quantum efficiency (EQE), whereas the responsivity and specific detection ratio under laser irradiation at 650 nm were 34.27 mA/W and 2.97 × 10^10^ Jones, respectively, and the photocurrent of the CsPbBr_3_ perovskite film photodetectors increased gradually with the increase in the incident optical power, while the response rate and specific detection rate decreased gradually. At the same time, in order to overcome the potential environmental hazards of lead-based materials, researchers have actively developed a series of lead-free perovskite oxide films with high functionality by PLD [183]. Hanani et al. [184] used PLD technology to epitaxially grow tin-doped BaTiO_3_ thin films (BaTi_0_._89_Sn_0_._11_O_3_, BTS) on SrTiO_3_ substrates deposited with LaNiO_3_ electrodes and studied their performance as relaxor ferroelectric (RFE) thin film capacitors. The results indicate that, under an electric field of 8.5 MV cm^−1^, the BTS film exhibits a high energy density of 105 J cm^−3^ and an energy efficiency of 80%, while, under an electric field of 6 MV cm^−1^, its quality factor can reach 1125 J cm^−3^. At the same time, BTS thin film capacitors can maintain excellent thermal stability and fatigue resistance within the temperature range of 0–140 ° C and after 1010 cycles of charging and discharging. In addition, Sheeraz et al. used PLD technology to epitaxially grow lead-free Bi_1/2_ (Na, K) _1/2_TiO_3_ (BNKT) composite films [185] and (K, Na) NbO_3_/La_0.7_Sr_0.3_MnO_3_ (KNN/LSMO) composite films [186] on the SrTiO_3_ (001) substrate and studied their ferroelectric and piezoelectric properties. The results showed that, by changing the contents of the incorporating cations (i.e., Bi, Na, and K ions) in the BNKT composite film, its crystal structure and ferroelectric properties could be controlled. The BNKT composite film deposited at a high temperature (570 °C) formed screw dislocations caused by off-stoichiometry-mediated strain relaxation, thereby exhibiting a pinched P−E hysteresis loop and a J–E curve with double-peak features. The KNN film with a layered structure (thickness of 35 nm) shows no significant polarization retention loss, while the KNN film with a columnar structure (thickness of 600 nm) exhibits significant polarization retention loss in the air environment, which may be caused by the pinning of charged domain walls by the protonation of hydrogen ions and/or the effective screening by polarization-bound charges, which are very attractive for designing two-dimensional thin film advanced devices.

Elemental doping is an important way to modify 2D perovskite films. Through PLD technology, the doping levels of elements can be precisely controlled while maintaining the perovskite structure, thereby effectively regulating the physicochemical properties of doped 2D perovskite films [187]. John et al. [188] used PLD technology to deposit BaSnO_3_ (BSN) films with different Ni doping levels (0, 1, 2, 3, 5, and 7 mol% Ni) and investigated their structures and properties. Their results showed that BSN films with different Ni doping concentrations exhibited a cubic-phase polycrystalline structure that preferentially grew along the (110) direction. Ni doping in BSN films was beneficial for improving their crystallinity, and when the Ni doping level reached 3 mol%, the BSN films exhibited the best crystal properties (Figure 10d), with a grain size and RMS roughness of 39 nm and 2.33 nm, respectively. Meanwhile, with the increase in Ni doping, the thickness of the BSN film is increasing, while the transmittance and bandgap energy are decreasing regularly (Figure 10e), but it still shows a transmittance of more than 80% in the visible-to-near-infrared region, which makes it possible to apply the BSN film in the field of optoelectronics. In addition, Hall effect measurements of the BSN films revealed that, with the increase in Ni doping, the carrier concentration of the BSN films increased by three orders of magnitude (2.98 × 10^11^ cm^−3^ to 3.59 × 10^14^ cm^−3^), and the mobility increased from 3.13 cm^2^V^−1^s^−1^ to 20.93 cm^2^V^−1^s^−1^, while the resistivity decreased by six orders of magnitude (4.05 × 10^9^ Ω cm to 1.13 × 10^3^Ω cm).

As an efficient preparation technology that can accurately design material composition and effectively maintain stoichiometry, PLD technology has shown distinctive advantages in the preparation of complex 2D perovskite films [189]. Kar et al. [190] used PLD technology to prepare Ba_0.95_Ca_0.05_Ti_0.95_Sn_0.05_O_3_/Ni_0.7_Zn_0.3_Fe_2_O_4_ (BCTSO/NZFO) bilayer composite magnetoelectric film materials with a perovskite-phase and cubic spinel-phase composite polycrystalline structure, where the grain size and root mean square surface roughness of the BCTSO layer were 80.90 nm and 4.97 nm, respectively, while those of the NZFO layer were 45.68 nm and 15.08 nm, respectively (Figure 10f). Meanwhile, the BCTSO/NZFO films exhibit a strong Maxwell–Wagner polarization effect, which results in a gradual decrease in their dielectric constant and loss tangent values with the increase in room temperature frequency (Figure 10g). In addition, the ferroelectric and magnetic properties of the BCTSO/NZFO composite films were analyzed and found to have excellent ferroelectric and subferromagnetic properties, with a maximum polarization of 6.8 μC/cm^2^, a residual polarization of 2.7 μC/cm^2^, a saturation magnetization of 86 emu/cc, and residual magnetization of 14.7 emu/cc. The BCTSO/NZFO composite film also exhibits strain-mediated direct magnetoelectric coupling, with a first-order magnetoelectric voltage coefficient of 124 mV/cm-Oe at room temperature and a maximum magnetoelectric voltage coefficient of 150 mV/cm-Oe (Figure 10h). Table 5 is the summary of the deposition conditions and properties of perovskite by PLD.

## 4. Advantages, Disadvantages, and Comparison with Other Technologies of PLD Technology

### 4.1. Advantages of PLD Technology

In summary, the PLD technology in high-quality 2D film material preparation and application of the current situation is summarized and analyzed, finding that it has the following advantages:

(1) Rich types and varieties of materials. PLD technology is mainly used to prepare various types of 2D film materials through laser ablation targets with high energy density, which do not have any limitations on the types and elemental composition of the target, and composite 2D film materials can also be constructed between different targets, which greatly increases the versatility and flexibility of the preparation of materials.
(2) Easy to maintain the stoichiometry of the target. In the process of PLD, due to the target being impacted by extremely high energy in a very short period of time, all the elements are rapidly deposited onto the substrate surface in the form of plasma plumes at almost the same rate, thus ensuring the stoichiometry of the prepared material is consistent with that of the target, which is very advantageous for preparing compounds with complex elemental compositions.
(3) The structure and morphology of the prepared materials can be controlled. In the process of preparing 2D film materials using PLD technology, various deposition parameters (laser energy density, background gas type and pressure, substrate material type, substrate temperature, etc.) can be adjusted to achieve control over the crystal structure, morphology, and size of the prepared products.
(4) Relatively low deposition temperature. Due to the high energy of the plasma plume generated by pulsed laser bombardment of the target, the deposition process does not require a high substrate temperature, and even at room temperature, it can be prepared in high-quality 2D film materials. This not only saves energy but also plays an important role in depositing 2D film materials on some substrates that do not tolerate high temperatures.
(5) Easy to operate and highly environmentally friendly. PLD technology does not involve chemical solvent reactions in the material preparation process, and the entire process almost does not produce pollutants, thus demonstrating excellent environmental friendliness in the process of use. At the same time, while ensuring the flexibility of process control, the operation is relatively simple, which lays the foundation for large-scale preparation of high-quality 2D film materials.

### 4.2. Disadvantages of PLD Technology

Just like the two sides of a coin, in addition to the above advantages, PLD also has some obvious disadvantages, which are as follows:

(1) Preparation of large-area materials is difficult. In PLD technology, the spot size of the pulsed laser is relatively small, which limits the cross-sectional area of the plasma plume formed by its impact on the target, thereby hindering the preparation of large-sized samples.
(2) Difficulty in precise control of material thickness and uniformity. The plasma plumes formed by different targets and laser parameters have different energies, and they diffuse, nucleate, and grow on the substrate at different deposition rates, thus seriously affecting the thickness and uniformity of the 2D material, which needs to be estimated and explored in multiple experiments.
(3) Deposition of target particles, splashing of plasma plumes, and defects in 2D film materials. When high-energy pulsed lasers bombard a target, they may cause a low-density target to deposit on the substrate in the form of small-sized large particles, thereby affecting the quality of 2D film materials. Meanwhile, plasma plumes with extremely high energy may cause the splashing of particles already deposited onto the substrate during the deposition process, thereby affecting the structure of 2D film materials. In addition, the transmission and interaction of plasma plumes in the background atmosphere are difficult to precisely control, which may form various defects in 2D film materials, thereby affecting their structures and properties.

### 4.3. Comparison of PLD Technology with Other Technologies in Terms of Performance

In order to clearly demonstrate the differences in performance between PLD technology and other competing technologies in preparing 2D film materials, we compared the characteristics of PLD technology with CVD, MBE, and ALD technologies in terms of cost, scalability, film uniformity, material crystallinity, large-area growth, and industrial feasibility. The results are shown in Table 6.

## 5. Conclusions and Outlook

The continuous discovery and innovation of materials actively promote the development of human society and the progress of science and technology. Among them, 2D film materials exhibit many novel physical properties due to their unique electronic structure and atomic arrangement and have a wide range of applications in high-tech fields such as optoelectronics, catalysis, solar cells, and sensors. Therefore, it is very urgent to find a suitable method and technology for the rapid and green preparation of high-quality 2D film materials. PLD not only has the characteristics of non-pollution and simple operation of the physical vapor phase method but also can maintain the stoichiometric ratio of the target during the preparation of 2D film materials, which has made it become the preferred method for the preparation of high-quality 2D film materials. Therefore, this paper provides a comprehensive and detailed review of the current status of PLD in the preparation of 2D film materials. PLD technology has achieved the preparation of high-quality 2D film materials in various compounds, such as carbides, sulfides, nitrides, oxides, and perovskites, and the physicochemical properties of 2D film materials can be effectively regulated by controlling the deposition parameters. Meanwhile, PLD technology can also be used to modify 2D film materials by means of element doping and heterojunction construction, thereby enhancing their properties and application value, especially in the scientific research field of complex perovskite thin film materials, which has made significant progress.

However, the limited large-scale preparation of materials, the difficulty of precise control of uniformity, the existence of material defects, poor repeatability, and outdated equipment integration seriously restrict the sustainable, high-quality, and industrialized development of PLD technology in the field of 2D material preparation. Therefore, in the future development of PLD technology, we should take more scientific and efficient measures to actively deal with the challenges and bottlenecks it faces in 2D material preparation.

(1) In-depth study of the mechanism of plasma plume generation, diffusion, adsorption, deposition, nucleation, and growth on the substrate during PLD. By combining PLD technology with in situ characterization techniques such as in situ X-ray photoelectron spectroscopy, in situ reflection high-energy electron diffraction, and in situ transmission electron microscopy, the growth process of 2D film materials can be monitored in real time, providing a practical and reliable basis for an in-depth understanding of the mechanism of PLD preparation of 2D film materials and optimization of the preparation process conditions.
(2) Actively explore the possibility of integrating PLD technology with other techniques for preparing 2D film materials, so as to overcome the problems of PLD itself, such as uneven thickness, multiple film defects, and limited scale of preparation, by complementing each other’s strengths and to provide new ways and opportunities for the preparation of higher-quality and larger-scale 2D film materials.
(3) Make full use of emerging technologies such as machine learning and molecular computing simulation to actively develop intelligent models that can simulate the deposition parameters of PLD, thereby more scientifically and quickly optimizing PLD deposition conditions and designing 2D film materials with novel structures and functions, and innovatively develop and enrich the high value-added applications of 2D film materials in the fields of quantum information and biomedicine.
(4) Scientifically construct and develop PLD processes suitable for large-scale production. Improve and prepare large molecular lasers with better energy density, thereby increasing the utilization rate of laser energy and the deposition rate of 2D film materials. At the same time, new PLD production processes such as continuous pulse laser deposition technology and multi-target synchronous deposition technology are developed and designed to improve the rate and quality of industrialized production. In addition, the synergistic cooperation between scientific research and enterprise production should be strengthened, and a new mechanism of joint innovation between industry, academia, and research should be established, so as to accelerate the industrialization process of PLD technology in the preparation of 2D film materials.

## Figures and Tables

**Table 1:** Summary of the deposition conditions and properties of carbides by PLD.

Materials	Substrates	Temperature	Background Gases	Pressure	Laser and Laser Energy	Properties	Reference
Graphene	Ni/SiO_2_	Room temperature (RT)	Vacuum	10^−7^ mbar	KrF 5 J/cm^2^	Ideal uniformity and low defect density.	[74]
Graphene	Ni/glass	750 °C	Vacuum	1 × 10^−5^ Torr	3.18 J/cm^2^	High-quality graphene films with high crystallinity.	[75]
Multilayer graphene	Si/SiO_2_	800 °C	Vacuum	3 × 10^−5^ Torr	KrF 1.5 J/cm^2^	A resistance of 6.6 k Ω.	[52]
Graphene/Ni	Si/SiO_2_	RT	Vacuum	2 × 10^−6^ Torr	KrF 100 mJ	The defects, surface coverage, and number of graphene layers can be controlled through modification of the magnetic field intensity.	[76]
Graphene-B	Ni/SiO_2_/Si	RT	Vacuum	10^−7^ mbar	KrF 6.2 J/cm^2^	Interfacial electron transfer constant 4.9 × 10^−3^ cm·s^−1^.	[77]
Graphene-N	SiO_2_/Si	780 °C	N_2_	9, 50, 100, and 240 Pa	KrF 100 mJ	The relative enhancement factor is about 2.5.	[78]
Graphene/xCu2O·yMnO	SiO_2_/Si	RT 300 C	N_2_	0.1 mbar	KrF 3 J/cm^2^	A more sensitive response to the test gases (NO_2_, O_3_, NH_3_, and H_2_S).	[78]
Graphene tilted Bragg fiber grating	Tilted Bragg fiber grating (TFBG)	_	Vacuum	2.9 × 10^−4^ Pa	Nd:YAG	Strain sensitivity is 0.48 nm/mε.	[79]
Reduced graphene oxide (rGO)	Silica wafer	680 °C	O_2_	0.5 mTorr	2.9 J/cm^2^	Transparency >97%, a resistivity of 30.3 mΩ cm, a carrier concentration of 2.3 × 10^13^ cm^−2^, and a mobility of 5.11 cm^2^V^−1^s^−1^.	[80]
Silica nanowires/graphene/zinc oxide nanoparticles	Silicon nanowires	_	Vacuum	10^−4^ mbar	Nd:YAG 700 mJ	Resistivity is 1.13 × 10^−2^ Ω.cm, carrier concentration is 3.92 × 10^18^ cm^−3^, Hall coefficient is 1.59 cm^3^C^−1^, the conductivity is 8.87 × 10^1^ Ω^−1^ cm^−1^, and mobility is 1.41 × 10^2^ cm^2^V^−1^s^−1^.	[81]
Ag/graphene /TiO_2_	Glass	200 °C, 300 °C	Vacuum	2 × 10^−5^ mbar	Nd:YAG 400 mJ, 600 mJ, 800 mJ, 900 mJ, 1000 mJ	Improve laser resonance.	[82]
DLC	SiO_2_/Si	700 °C	Vacuum	1 × 10^−3^ Pa	Nd:YAG 5 J/cm^2^	Specific resistance is 1.5 × 10^−3^ Ω·m.	[83]
DLC	Intrinsic silicon	RT	Vacuum	8–12 × 10^−4^ Pa	KrF 300 mJ, 400 mJ, 500 mJ	High infrared transmittance and refractive index and low extinction coefficient. The	[84]
DLC	Si	300 °C	Vacuum	1.5 × 10 ^−6^ Torr	KrF 1.2 J/cm^2^	maximum area-specific capacitance is 48.25 mF/cm^2^, and the capacitance retention and the coulombic efficiency after 5000 cycles were 97.3% and 93.5%.	[85]
DLC	SiO_2_ /Si	RT	Vacuum	10–8 mbar	KrF 0.2 J/cm^2^	Hydrogen-free DLC layers with a high level of sp^3^ hybridization are obtained.	[86]
SWCNTs, MWCNTs	Silicon wafer	_	Vacuum	2.5 × 10^−2^ mbar	Nd:YAG 700mJ	Short-circuit current density of SWCNT cells is 30 mA/cm^2^, and power conversion efficiency is 5.6%.	[87]
SWCNTs	Carbon fibres	100 °C–565 °C	_	_	KrF 600 mJ	A 20% increase in interfacial shear strength (IFSS) was observed.	[88]

**Table 2:** Summary of the deposition conditions and properties of sulfides by PLD.

Materials	Substrates	Temperature	Background Gases	Pressure	Laser and Laser Energy	Properties	Reference
MoS_2_	c-Al_2_O_3_	800 °C	Vacuum	5 × 10^−6^ mbar	KrF 4 J/cm^2^	Bandgap = 1.86 eV, photocurrent = 0.32 nA, and photoresponse = 3 mAW^−1^.	[108]
MoS_2_	Silicon, Quartz	400 °C	Vacuum	5 × 10^−4^ Pa	KrF 70 J/cm^2^ 80 J/cm^2^ 90 J/cm^2^ 100 J/cm^2^ 110 J/cm^2^	Direct optical band gap = 1.614 eV. opening voltage = 0.61 V, and rectification ratio = 457.0.	[109]
MoS_2_ nanoribbons	Al_2_O_3_	700 °C	Vacuum	6 × 10^−7^ mbar	KrF 2 J/cm^2^	Responsivity of 8.72 × 10^2^ AW−1 at 532 nm.	[110]
MoS_2_-Li	c-Al_2_O_3_	600 °C	_	-	KrF	Resistance and activation energy of MoS_2_-Li film rapidly increase.	[111]
MoS_2_-Ag	Si/SiO_2_ wafer	700 °C	Vacuum	10^−6^ mbar	Nd:YAG	Enhancement factor of 1.8 × 10^8^ and detection limit of 10^−12^ M for the R6G.	[112]
MoS_2_/SrRuO_3_(111)	c-Al_2_O_3_	700 °C	O_2_	100 mTorr	KrF 3 J/cm^2^	Room temperature resistivity of 1.83 and 1.39 μΩ for FL and BL MoS_2_/SrRuO_3_.	[113]
WS_2_/MoS_2_	Au /mica	RT	_	_	KrF 2 J/cm^2^	A strong interlayer coupling between MoS_2_ and WS_2_.	[114]
CeO_2_/MoS_2_/WSe_2_	SiO_2_/Si	500 °C	Ar	100 mTorr	KrF 1.2 J/cm^2^	Higher tunneling current.	[115]
MoS_2_/GaN heterojunction	GaN/c-Al_2_O_3_	860 C	Vacuum	2 × 10^−6^ Torr	KrF 70 mJ	Schottky barrier height of 0.36 eV.	[116]
Si/MoS_2_	Al alloy	_	Vacuum	10^−4^ Pa	Nd:YAG 2.5 J	Si/MoS_2_ coating possesses a self-lubricating property.	[117]
WS_2_	Glass	180 °C	Vacuum	3 × 10^−6^ torr	Nd:YAG	Direct bandgap of 1.98 eV and a defect energy of 1.28 eV.	[118]
WS_2_	Si/SiO_2_	700 °C	Vacuum	10^−5^ mbar	Nd:YAG 190 mW	Increased saturation magnetization.	[119]
WS_2_, WS_2_-Ag	Silicon	400 °C	Vacuum	4 × 10^−6^ mbar.	Nd:YAG 80 mJ	The detection limits of Ag-WS_2_ for RhB and MO dyes are 10^−16^ M and 10^−17^ M. Raman enhancement factor distributions of 1.0 × 10^8^ and 2.6 × 10^7^.	[120]
WS_2_/WO_3_ heterostructure	Amorphous Al_2_O_3_	_	Vacuum	1.5 × 10^−8^ Torr	KrF	The fast decay time of WS_2_/WO_3_ is 1.0 μs, and the slow decay time is 4.1 μs.	[121]
SnS	Quartz, Silicon wafer	_	Vacuum	10^−4^ Torr	Nd:YAG 700 mJ	Spectral sensitivity, specific detectability, and quantum efficiency are 0.72 A/W, 2.85 × 10^11^cm·Hz^1/2^·W^−1^, and 70.18%, respectively.	[122]
CdS	Si wafer	_	Vacuum	5 × 10^−6^ mbar	Nd:YAG 140 mJ 180 mJ 220 mJ	External quantum efficiency value = 129.4%, the response time = 179 ms, and recovery time = 161 ms.	[123]
NiS_2_/MnS and MnS/NiS_2_ heterojunction	FTO	500 °C	O_2_	5 Pa	350 mJ	Power conversion efficiency (PCE) could reach 6.44%.	[124]
ZnIn_2_S_4_ (ZIS)	SiO_2_/Si	450 °C	Vacuum	6 × 10^−4^ Pa	KrF	Responsivity, external quantum efficiency, and detection rate were 1.4 AW, 430%, and 9.8 × 10^9^ Jones (1 Jones = 1 cm Hz^1/2^ W^−1^).	[125]
Cu_2_ZnSnS_4_ (CZTS)	SiO_2_/Si sapphire	RT to 500 °C	_	_	KrF 0.8 to 2.4 J/cm^2^	The composition of CZTS films changed continuously with different deposition temperatures.	[126]
Cu_2_ZnSnS_4_	modified substrate with MoSe_2_	RT, 200 °C 250 °C 300 °C 350 °C	Vacuum	10^−5^ Torr	KrF 270 mJ	Conversion efficiency. Conversion of CZTS devices is 13.99%.	[66]
SnS-Ag, SnS-Pd	SiO2/Si	RT to 500 °C	Vacuum	1 × 10^−6^ mbar	Nd:YAG 90 mJ	The response of SnS-Ag is 138% toward 2 ppm NO_2_, the response of SnS-Pd is 55% toward 70 ppm H_2_, and limit of detection (LOD) < 1 ppm.	[127]

**Table 3:** Summary of the deposition conditions and properties of oxides by PLD.

Materials	Substrates	Temperature	Background Gases	Pressure	Laser and Laser Energy	Properties	Reference
ZnO	Porous silicon Quartz	_	Vacuum	5 × 10^−2^ mbar	Nd:YAG 400, 600, 800 mJ/pulse	Optical energy gap = from 3.44 to 3.79 eV, ideal factor = 2.33, and EQE = 92.31%.	[144]
ZnO-Cu (3 wt%, 5 wt% and 7 wt% Cu)	Fused silica	300 °C	O_2_	1 mTorr	Nd:YAG 1 J/pulse	Optical band gap energy decreases from 3.26 eV to 3.0 eV with increasing the Cu concentration from 0 to 7 wt%.	[145]
ZnO-Sn	Silicon	RT 400 °C	O_2_	100 mTor 700 mTorr	Nd:YAG 12.92 J/cm^2^	Exhibited the most pronounced antibacterial activity.	[146]
ZnO-Ni (3 wt%, 5 wt% and 7 wt% Cu)	Fused silica	300 °C	O_2_	1 mTorr	Nd:YAG 1 J/pulse	The effective atomic mass and lattice parameters changed.	[147]
ZnO-N	Fused silica	RT	N_2_/O_2_	_	Nd:YAG 16 J/cm^2^	Cc = 5.3 × 10^18^ cm^−3^, resistivity = 2 Ωcm, and Cm = 0.5 cm^2^/V·s.	[148]
Pd/SnO_2_	Quartz	RT	O_2_	100 mTor 700 mTorr	Nd-YVO4 0.2 W	The sensitivity of this sensor (Pd/SnO2) = 0.21 Hz/ppm, and LOD = 142 ppm.	[149]
SnO_x_	ITO-coated glass	150 °C	O_2_/Ar	5 × 10^−3^ mbar	KrF 1.5−1.6 J/cm^2^	Power conversion efficiencies > 18%.	[150]
SnO_2_	Stainless steel	300 °C, 400 °C 500 °C	O_2_	300 mTorr	KrF 350 mJ	Specific capacity = 488 mAh/g and coulombic efficiency = 96%.	[151]
SnO_2_/ RuO_2_	304 stainless steel	150 °C	O_2_	150 mTorr	KrF 300 mJ	Specific capacitance = 170.2 Fg^−1^, capacitive retention of 81.27% over 10,000 cycles, energy density = 19.05 Wh/kg, and power density = 645 W/kg.	[152]
SnO_2_/ TiO_2_	FTO	400 °C	O_2_	55 mTorr	KrF 250 mJ	Cm = 6.710 cm^2^/V·s and resistivity = 1.213 × 10^−2^ Ωcm. Hall coefficient = 31.86 × 10^−2^ cm^−3^C^−1^) and conductivity = 82.41 (Ωcm)^−1^.	[153]
Ag/CuO	Quartz	_	Vacuum	10^−4^ torr	Nd:YAG	Optical transmittance = 96% and direct band gap = 2.15 eV.	[154]
Ag/CuO	Quartz	_	Vacuum	10^−4^ torr	Nd:YAG	Optical transmittance = 97%, direct band gap = 2.43 eV, and outstanding catalytic efficiency for the degradation of 4-nitrophenol.	[155]
CdO CuO/CdO	Quartz	_	Vacuum	10^−4^ torr	Nd:YAG	The transmittance = 96%, direct optical band energy band gap for CdO = 2.41 eV, and direct optical band energy band gap for CuO/CdO = 3.39 eV.	[156]
CuO	MgAl_2_O_4_ (110)	400 °C	Oxygen plasma	0.09–0.8 Pa.	KrF 4 J/cm^2^	The optical band gap = 1.14 to 1.47eV.	[157]
Cu_2_O, CuO	Glass	380 °C	N_2_/O_2_	0.2 Pa and 2 Pa	Nd:YAG 10 J/cm^2^	The direct band gap for Cu_2_O = 2.45 eV and CuO = 2.25 eV. Photocatalytic efficiencies for MB dye degradation > 96%.	[158]
Nd_2_O_3_/CuO	Silicon	RT	Vacuum	2.2 × 10^−2^ kPa.	200 mJ	Sensitivity = 180% against 79 ppm NH_3_ at a working temperature of 50 °C.	[159]
Co-Fe-B-O	FTO	_	Argon atmosphere	1.5 × 10^–2^ mbar	KrF 3 J/cm^2^	Overpotential of 315 mV at 10 mA/cm^2^ and Tafel slope of 31.5 mV/dec.	[160]
Co_3_O_4_/WO_3_	Ni foam Carbon paper Si wafer FTO	500 °C. 650 °C	Vacuum	10^−6^ mbar	KrF 0.8 J/pulse	Volumetric capacitance = 141.9 F cm^−3^ and the voltage window = 1.6 V. Coulombic efficiency > 97%, and capacitance retentions = 91% in cycle life 27,000.	[161]

**Table 4:** Summary of the deposition conditions and properties of h-BN by PLD.

Materials	Substrates	Temperature	Background Gases	Pressure	Laser and Laser Energy	Properties	Reference
h-BN	Stainless steel	600 °C	Ar: N_2_ = 3:1	10 mTorr	4 J/cm^2^	Current density = 13.2 nA/cm^2^, corrosion rate = 11.7 × 10^−3^ mm/y, and corrosion resistance = 6.28 × 10^6^ Ω cm^2.^	[169]
h-BN	c-Al_2_O_3_	800–1250 °C	N_2_	10–300 mTorr	KrF 300–700 mJ	On/off ratio of >10^4^, high photoresponsivity, and a sharp cut-off wavelength of 220 nm.	[175]
h-BN	silicon wafer	600 °C	–	–	2.4 mJ	Hardness = 2.47 ± 0.20 GPa. The elastic modulus = 74.32 ± 7.98 GPa.	[176]
h-BN	SiC	750 °C	N_2_	100 mTorr	KrF 2.3 mJ/cm^2^	Cross-plane thermal conductivity from 1.5 to 0.2 W/(m K), and the thermal boundary conductance interface = 22.3–47.5 MW/(m^2^ K).	[57]
h-BN	c-Al_2_O_3_ (0001)	–	N_2_	100 mTorr	KrF 2.2 J/cm^2^	Contact angle increased from 55° to 60°, friction coefficient = 0.0002, and refractive indices = 1.53–1.55.	[167]
w-BN	c-Al_2_O_3_	400 °C 800 °C	Vacuum	10^−5^ Torr	–	The hardness = 37 GPa, and the elastic modulus = 339 GPa.	[171]
Carbon-doped h-BN	Si/SiO_2_ Mo	300 °C	CH_4_	200 mTorr	2 × 10^8^ Wcm^−2^	The hysteresis loop’s Schottky diodes are directly affected by the parameters of resistance and barrier lowering.	[173]
h-BN	Li-pellet	–	Ar	50 mTorr	KrF 2 J/cm^2^	Effectively improving the constant current cycling life of lithium metal batteries (more than 1800 h) and reducing their electrochemical impedance.	[174]

EQE = external quantum efficiency, Cc = carrier concentration, Cm = carrier mobility, and LOD = limit of detection.

**Table 5:** Summary of the deposition conditions and properties of perovskite by PLD.

Materials	Substrates	Temperature	Background Gases	Pressure	Laser and Laser Energy	Properties	Reference
SrMnO_3_	FTO-coated glass	600 °C	O_2_	100 mTorr	KrF 300 mJ	Cc = 1.37 × 10^12^ cm^−3^, Cdu = 5.56 × 10^−6^ S/cm, and Cm = 24.5 cm^2^/V·s.	[181]
CsPbI_3_	Glass Silicon	600 °C	Ar	5 × 10^−4^ mbar	Nd:YAG 5.7 J/cm^2^ 7.1 J/cm^2^ 8.5 J/cm^2^ 10 J/cm^2^	Bandgap 1.83 eV, Cc = 1.38 × 10^12^ cm^−3^, Cm = 164.7 cm^2^/V·s, Rp = 8 A/W, SDR = 10^14^ Jones, and EQE = 17.5 × 10^2^%.	[191]
CaTiS_3_	Al_2_O_3_	600 °C	Vacuum	5 × 10^−6^ mbar 1 × 10^−5^ mbar	KrF 1–2 J/cm^2^	The absorption coefficient is in the 10^5^ cm ^−1^ range, the direct bandgap = 1.59 eV, and Cc ~10^22^ cm^−3^ range.	[192]
p-Si/n-CsPbBr_3_	Ingle crystal silico	200 °C	Vacuum	10^−3^ Pa	KrF 350 mJ	Rp = 780 mA/W, and SDR = 6.78 × 10^11^ Jones.	[62]
LaScO_3_/SrTiO_3_	(001) SrTiO_3_	600 °C to 800 °C	O_2_	10^−6^ mbar 10^−4^ mbar	KrF 1.5 to 2.5 J/cm^2^	The carrier density is 10^14^ to 10^15^ e^−^ cm^−2^, Rv = of 5.2 kΩ, and Cm = 8.5 cm^2^/V·s.	[193]
LaVO_3_/ SrTiO_3_	DyScO3 (101)	900 °C	0.1% O_2_/99.9%N_2_ gas mixture	2 mTorr	KrF	Bandgap 1.49 eV, Cc = 1.4 × 10^18^ cm^−3^, and Cm = 70 cm^2^/V·s.	[194]
BaSnO_3_- Ni (1, 2, 3, 5, and 7 mol %)	Quartz	600 °C	O_2_	0.02 mbar	Nd:YAG 60 mJ	Cc = 2.98 × 10^11^–3.50 × 10^14^ cm^3^, Cm = 3.13–20.93 cm^2^/V·s, and ER = 4.05 × 10^9^–1.13 × 10^3^ Ω cm.	[188]
BaTiO_3_-S	SrTiO_3_ (001)	640 °C	O_2_	100 mTorr	KrF 1.2 J/cm^2^	Pr = 23.15 µC/cm^2^ and Ps = 35.69 µC/cm^2^.	[195]
BaSnO_3_-La (5%)	Sapphire (0001)	700 °C	O_2_	5 × 10^−2^ mbar	Nd:YAG 240 J/cm^2^.	Transmissions = 65–75% and optical bang gap = 3.22–3.51 eV.	[196]
Ba_0.95_Ca_0.05_Ti_0.95_Sn_0.05_O_3_/Ni_0.7_Zn_0.3_Fe_2_O_4_	Pt (111)/TiO_2_/SiO_2_/Si	700 °C 650°C	O_2_	150 mTorr	KrF 1.5 J/cm^2^	A maximum polarization = 6.8 µC/cm^2^, Pr = 2.7 µC/cm^2^, and Ms = 86 emu/cc. Mr = 14.7 emu/cc.	[190]
Ba_0:6_Sr_0:4_TiO_3_	_	_	Vacuum	10^−3^ Torr	Nd:YAG 700 J/cm^2^	Dielectric constant = 420, dissipation factor = 0.04,	[197]
La_0.67_Sr_0.33_MnO_3_	(001) SrTiO_3_	750 °C	O_2_	15 mTorr	_	damping value = 0.0014, and damping change rate = 210%.	[198]
Pb_0.92_La_0.08_(Zr_0.52_Ti_0.48_)O_3_/YBa_2_Cu_3_O_7-__ ẟ _	(00l) LaAlO_3_	800 °C 650 °C	O_2_	30 Pa	_	Good superconductivity, and its critical transition temperature is ~87 K. Pr = 21 µC/cm^2^.	[199]

Cc = concentration of charge carriers, Cdu = conductivity, Cm = carrier mobility, Rp = responsivity, SDR = specific detection ratio, Er = electrical resistivity, EQE = external quantum efficiency, Rv = resistance value, Pr = polarizations remnant, Ps = polarization saturated, Ms = saturation magnetization, and Mr = remnant magnetization.

**Table 6:** Comparison of the characteristics between PLD technology and CVD, MBE, and ALD technologies.

Parameter	PLD	CVD	MBE	ALD
Cost	High	Varies by type	Extremely expensive	Relatively high
Scalability	Poor	Good	Limited	Relatively good
Film uniformity	Good uniformity in small areas, but poor uniformity on large-area substrates	Good uniformity, but for films with complex-shaped substrates or high aspect ratio structures, the uniformity is poor	Capable of achieving atomic-level precise control, and excellent film uniformity and structural integrity	Highly uniform films can be grown on substrates of various shapes, with thickness control accuracy reaching sub-nanometer levels
Material crystallinity	High crystalline quality two-dimensional materials can be prepared	By optimizing process parameters, high crystallinity films can be prepared	High-quality single-crystal films at the atomic level, high crystallinity of the material	Good crystallinity can be obtained, and atoms can be arranged in an orderly manner on the substrate
Large-area growth and Industrial feasibility	Uniform growth of centimeter-level area. It is suitable for preparing special and complex film materials. Its application in large-scale industrial production is relatively limited and mainly used in high-end fields.	By optimizing processes and equipment, large-area uniform growth can be achieved, which is widely used in fields such as integrated circuit manufacturing, solar cells, and flat panel displays, with high industrial feasibility	Small area high-quality growth can be achieved in the laboratory, but the technical difficulty and cost of achieving large-area growth are high. It is mainly used in high-end semiconductor research and production fields, and the industrial application scope is relatively narrow	Uniform films can be grown on large-area substrates, but the deposition rate is slow, and the efficiency of large-area growth is relatively low. It is widely used in advanced semiconductor manufacturing and nanotechnology, especially suitable for preparing ultra-thin and highly uniform films.
